# Direct Population of Triplet States for Efficient Organic Afterglow through the Intra/Intermolecular Heavy-Atom Effect

**DOI:** 10.3390/molecules29051014

**Published:** 2024-02-26

**Authors:** Jie Yuan, Yongrong Wang, Binbin Zhou, Wenjing Xie, Botao Zheng, Jingyu Zhang, Ping Li, Tian Yu, Yuanyuan Qi, Ye Tao, Runfeng Chen

**Affiliations:** 1 Engineering Technology Training Center, Nanjing Vocational University of Industry Technology, 1 Yangshan North Road, Nanjing 210023, China; shuipingyuanjie@niit.edu.cn (J.Y.); 2020101101@niit.edu.cn (B.Z.); WhitneyXie8894@outlook.com (W.X.); 2015100812@niit.edu.cn (T.Y.); 2 State Key Laboratory of Organic Electronics and Information Displays & Institute of Advanced Materials (IAM), Nanjing University of Posts & Telecommunications, 9 Wenyuan Road, Nanjing 210023, China; iamyrwang@163.com (Y.W.); 15934162709@163.com (B.Z.); sxxyzjy@126.com (J.Z.); iampingli@njupt.edu.cn (P.L.); iamyyqi@njupt.edu.cn (Y.Q.)

**Keywords:** organic afterglow, S_0_→T_1_ absorption, heavy atom effect, visible-light excitable, pattern encryption

## Abstract

Organic afterglow is a fascinating phenomenon with exceptional applications. However, it encounters challenges such as low intensity and efficiency, and typically requires UV-light excitation and facile intersystem crossing (ISC) due to its spin-forbidden nature. Here, we develop a novel strategy that bypasses the conventional ISC pathway by promoting singlet-triplet transition through the synergistic effects of the intra/intermolecular heavy-atom effect in aromatic crystals, enabling the direct population of triplet excited states from the ground state. The resulting materials exhibit a bright organic afterglow with a remarkably enhanced quantum efficiency of up to 5.81%, and a significantly increased organic afterglow lifetime of up to 157 microseconds under visible light. Moreover, given the high-efficiency visible-light excitable organic afterglow emission, the potential application is demonstrated in lifetime-resolved, color-encoded, and excitation wavelength-dependent pattern encryption. This work demonstrates the importance of the direct population method in enhancing the organic afterglow performance and red-shifting the excitation wavelength, and provides crucial insights for advancing organic optoelectronic technologies that involve triplet states.

## 1. Introduction

**Figure 1:**
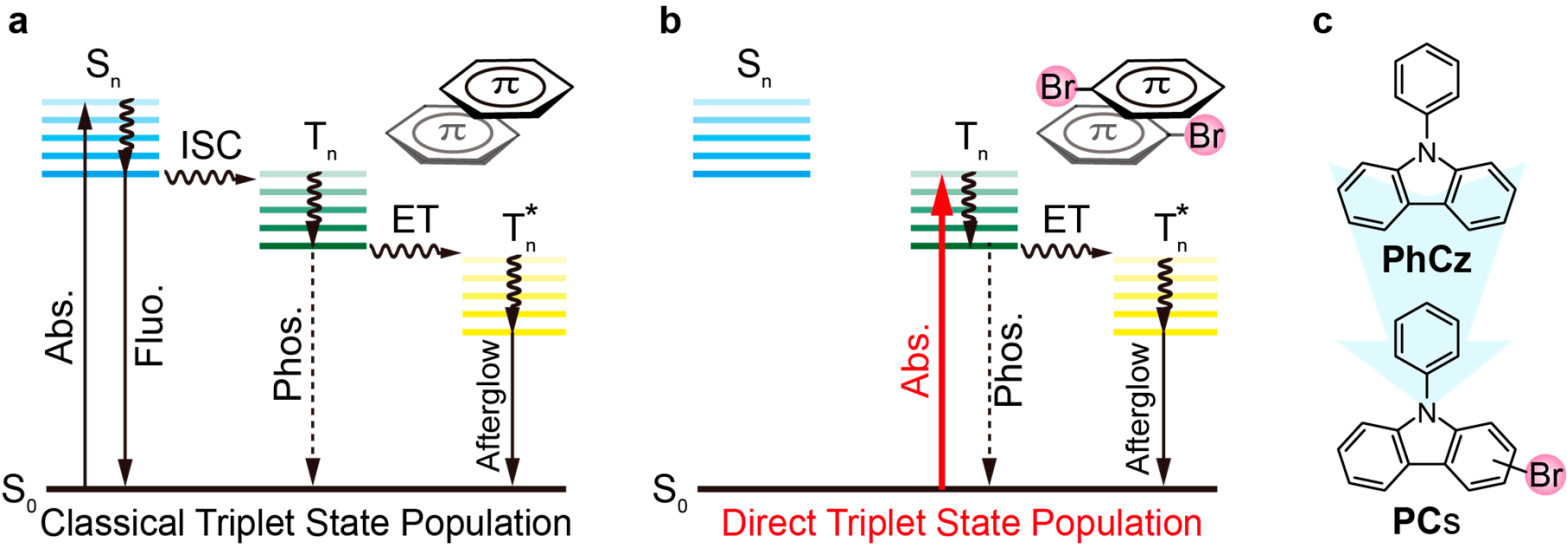
Two distinct approaches to achieve organic afterglow. (**a**) The traditional mechanism; (**b**) direct population of the T_1_ state through intra/intermolecular heavy-atom effect-induced S_0_→T_1_ absorption; and (**c**) the corresponding molecular design strategy.

Organic afterglow, renowned for its distinctive mechanism of photoelectron storage and release, has recently garnered tremendous attention in various fields, including spectroscopy, photonics, and materials science [1,2,3]. Additionally, it has been widely acknowledged that organic afterglow is a kind of room-temperature phosphorescence due to the radiative decay of the triplet exciton. This is fundamentally distinct from thermally activated delayed fluorescence, which involves fluorescence due to the radiative decay of the singlet exciton [3,4]. In recent years, various organic afterglow phenomena have been observed, including single-component purely organic aromatic molecules [5], multi-component host–guest/exciplex systems [6], polymers [7], clusters [8], carbon dots [9], and metal–organic frameworks [10], and more and more strategies have been developed for the manipulation of the morphology, color, operational wavebands, and persistent duration [11,12,13]. These sought-after accomplishments have sparked a growing interest in the design of optical sensors, photocatalysts, detectors, bio-labels, and photonic devices [14,15,16,17]. Specifically, there are two general approaches to designing organic afterglow materials (Figure 1a). The first approach involves promoting intersystem crossing (ISC) to efficiently populate triplet excited states. This is achieved through the introduction of aromatic aldehyde groups, carbonyl groups, or heteroatoms [18,19]. The second approach focuses on enhancing intra/intermolecular interactions to inhibit nonradiative transitions, allowing the gradual release of excitons to the ground state (S_0_). This is accomplished through the utilization of H-aggregation, crystallization, hydrogen bonds, or a rigid matrix [20,21,22,23]. However, organic afterglow suffers from low luminescent intensity and efficiency, and often requires UV-light excitation, which has harmful effects on living organisms [24]. These limitations significantly impede the development and applications of purely organic afterglow molecules. In 2019, Mao et al. introduced an innovative design method utilizing two-photon absorption, leading to a noticeable organic afterglow emission triggered by a near-infrared laser (808 nm) under ambient conditions [25]. Nevertheless, high ISC is still needed to populate triplet excited states (T_n_) from the lowest singlet excited state (S_1_) upon photoexcitation, which is usually difficult to realize in metal-free organic molecules.

Recognizing that efficient phosphorescence due to the spin-forbidden T_1_→S_0_ transition has been widely recognized [2,3,22], the reverse process of S_0_→T_1_ transition could also effectively populate T_1_ without the participation of the S_1_→T_n_ ISC pathway (Figure 1b). However, it is exceedingly weak in most organic materials, except for certain metal complexes and aromatic molecules [26,27,28]. In 2019, we demonstrated the significant potential of this direct population method in developing highly efficient organic afterglow materials for the first time [29]. This approach not only enhances organic afterglow efficiency by eliminating the involvement of high-lying excited states and unwanted relaxation processes, but also red-shifts the excitation wavelength, resulting in an intense organic afterglow emission with high quantum efficiency under visible-light excitation [28]. This advancement holds significant promise for expanding the application of organic afterglow materials in the biological field, particularly in bio-imaging. Maybe due to the notably lower cross-section compared to the spin-allowed excitation via the S_0_→S_1_ transition, only a limited number of organic afterglow molecules based on this strategy have been reported. Nevertheless, the strategy of bypassing the traditional S_1_→T_n_ ISC to directly populate triplet excited states via S_0_→T_1_ absorption still holds promise.

In this study, we have successfully designed a series of organic afterglow molecules capable of efficient direct S_0_→T_1_ absorption by incorporating heavy-atom bromine into a molecule of 9-phenyl-9H-carbazole (**PhCz**) (Figure 1c). The introduction of the bromine atom induces a combined and synergetic intra/intermolecular heavy-atom effect, effectively stimulating enhanced S_0_→T_1_ transition in these Br-substituted aromatic molecules. Notably, this direct S_0_→T_1_ absorption strategy significantly improves afterglow properties, resulting in an enhanced efficiency of up to 5.81% and an elongated emission lifetime of up to 157 microseconds (ms) under visible light excitation at 400 nm. This represents a substantial improvement, being up to 15 times more in efficiency and with a 2.5 times longer emission lifetime compared to under UV irradiation at 295 nm. These findings underscore the remarkable advantages of S_0_→T_1_ absorption in metal-free organic materials for directly populating triplet states, thereby promoting the development of a highly efficient and long-lived organic afterglow. Additionally, a flexible pattern encryption device that relies on efficient visible-light excitable organic afterglow materials was developed, incorporating lifetime-resolved, color-encoded, and excitation wavelength-dependent features. The insights gained from this research provide fundamental understandings and valuable guidelines for the exploration and applications of direct triplet state population-involved organic optoelectronic materials.

## 2. Results and Discussion

### 2.1. Molecular Design and Synthesis

**Figure 2:**
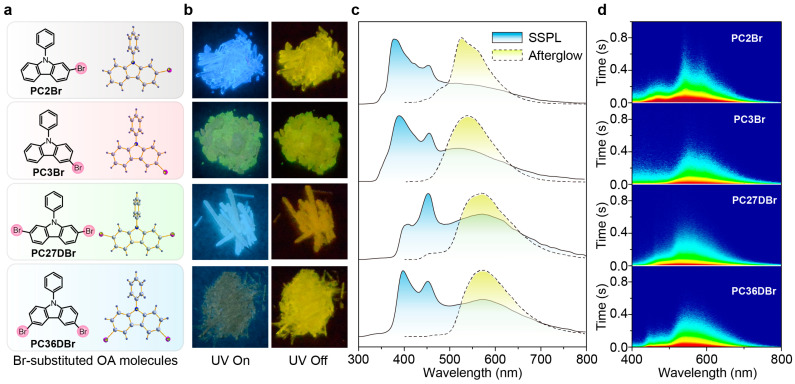
Designing organic afterglow molecules through the direct population of triplet excited states. (**a**) Br-substituted organic afterglow molecules with corresponding single-crystal structures; (**b**) photographs before (**left**) and after (**right**) 365 nm excitation; (**c**) SSPL and organic afterglow emission spectra; and (**d**) transient PL decay images excited by 295 nm light at room temperature.

**Scheme 1:**
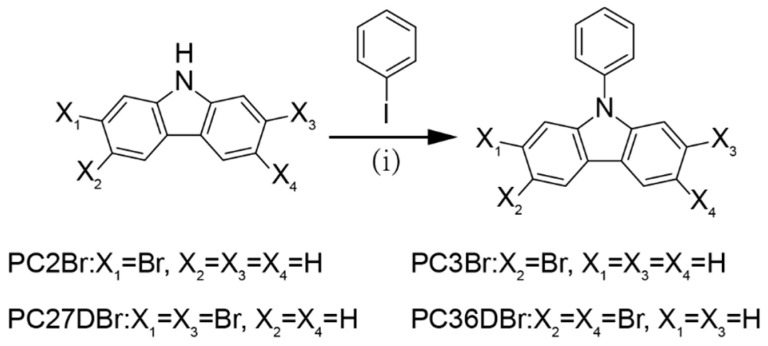
Synthetic route of the molecules of **PC2Br**, **PC3Br**, **PC27DBr**, and **PC36DBr**: (i) iodobenzene, copper iodide, K_2_CO_3_, N, N-Dimethylformamide, 180 °C, 24 h.

To facilitate S_0_→T_1_ absorption, the intra/intermolecular heavy-atom effect must be engaged and synergistically combined in optoelectronically active π-conjugated systems with efficient internal interactions in single molecular states and external effects in crystals. In contrast to previous methods, we directly incorporated heavy atoms into the carbazole chromophore moiety, thereby enhancing both the intra- and inter-molecular heavy-atom effects in the weak organic afterglow molecule **PhCz**, resulting in the design of heavy-atom incorporated organic aromatic afterglow molecules named **PC2Br**, **PC3Br**, **PC27DBr**, and **PC36DBr** (Figure 2a). Their synthesis proved to be convenient and efficient using a one-step Ullman reaction, yielding high efficiencies ranging from 65% to 90% (Scheme 1) [30]. Detailed information on the synthetic procedures, molecular structure characterizations, material purities, and single-crystal structures can be found in the Supporting Information (Figures S1–S13 and Table S1). Liquid chromatograph mass spectrometer (LC-MS) data were generated to demonstrate the material purities of 99.87%, 99.16%, 100.00%, and 99.59% for the final products, and no isomers of carbazole or bromocarbazole were found, which significantly mitigates any afterglow emission issues caused by purity [31].

### 2.2. Photophysical Property Investigation

The Br-substituted **PhCz** derivates (**PC**s) exhibit very similar ultraviolet-visible (UV-Vis) absorption spectra to those of **PhCz** in dilute solutions and thin films, and the weak absorption bands around 345 nm, stemming from the n–π* transitions of the carbazole moiety, are capable of enhancing ISC [20]. Characteristic fluorescent emission bands of the carbazole moiety were observed in the photoluminescence (PL) spectra of these compounds in both solution and films (Figures S14–S16). Interestingly, when these molecules formed crystals, extraordinary organic afterglow emission became apparent (Figure 2b). Accompanied by the fluorescence bands, the organic afterglow emission peaks around 560 nm can even be observed in the steady-state PL (SSPL) spectra (Figure 2c and Figure S17). After bromine substitution, the efficiency and emission lifetime of organic afterglow significantly decreased compared to **PhCz** under UV-light (295 nm) excitation due to the enhancement of the spin-orbit coupling (SOC) ability (Figure 2d and Table 1) [32]. However, when these crystals were excited by 400 nm, stronger afterglow emissions with higher efficiencies were observed in the Br-substituted compounds compared to UV-light excitation (Table 1 and Figures S18 and S19).

Excitation spectra of the organic afterglow peaks were studied to understand the remarkably efficient visible light-excited afterglow emission, which demonstrated an enhanced organic afterglow efficiency and an elongated lifetime compared to the organic afterglow emission excited at 295 nm [33]. The **PC**s proved to be efficiently photoexcited by visible light, with the wavelength ranging from 350 to 470 nm under ambient conditions. This phenomenon is particularly evident in the **PC36DBr** crystal, exhibiting significantly strengthened afterglow peaks when the excitation wavelength is longer than 400 nm at room temperature. (Figure S20).

**Figure 3:**
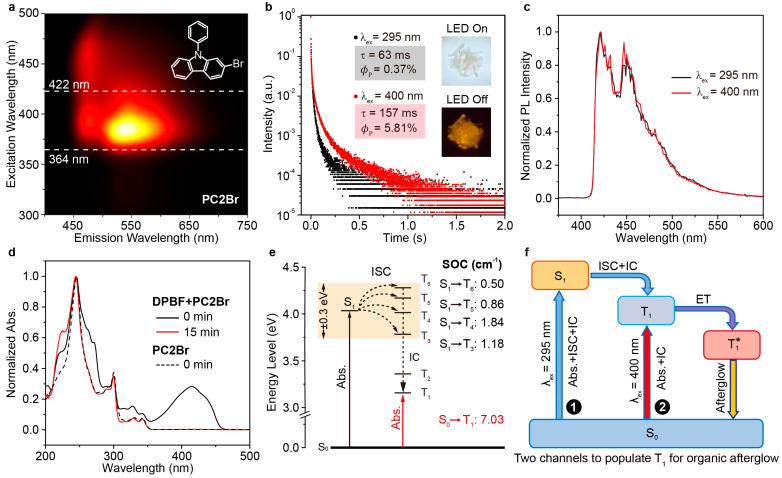
UV- and visible light-excited organic afterglow of **PC2Br** crystals. (**a**) Phosphorescence excitation–emission mapping and (**b**) lifetime decay profiles excited at 295 nm and 400 nm at room temperature, with insets showing individual PL photographs (above) and afterglow images (below) under LED excitation. (**c**) Phosphorescence spectra in 2-Me THF at 77 K with a delay time of 10 ms after the 295 (black line) and 400 nm (red line) excitation. (**d**) Absorption spectra of **PC2Br** (20 μM, upper, dashed line) and its mixtures with **DPBF** (50 μM, solid line) in THF after excitation at 400 nm for 0 (black line) and 15 min (red line). (**e**) TD-DFT-calculated energy levels and SOC values of the singlet and triplet excited states of **PC2Br**, and (**f**) organic afterglow through two pathways.

Taking the **PC2Br** crystal as a representative example, we initially conducted phosphorescence excitation–emission mapping with a delay time of 10 ms to investigate the remarkably efficient visible light-excited organic afterglow emission [34]. The organic afterglow emission around 530 nm appeared weak under UV light (295 nm) excitation but experienced a significant enhancement under visible light. Notably, the afterglow peaks were substantially strengthened when the excitation wavelength ranged from 364 to 422 nm in the **PC2Br** crystal at room temperature (Figure 3a). This enhancement resulted in an organic afterglow efficiency 15 times higher and an elongated lifetime 2.5 times longer compared to the 295 nm excited organic afterglow emission. The brominated compound **PC2Br** proves to be effectively photoexcited by visible light, extending to the point of being responsive to white LED light at room temperature (Figure 3b and Figure S21).

Is the organic afterglow emission effectively excited by visible light, even at longer wavelengths than their fluorescence peaks in solution and film states (345~365 nm)? To comprehend this phenomenon, we examined the phosphorescence spectra of brominated compounds in 2-methyl tetrahydrofuran (2-Me THF) solution at 77 K with a delay time of 10 ms (Figure S22). Under UV-light excitation at 295 nm, a phosphorescence spectrum similar to typical triplet features of carbazole was observed for **PC2Br**, with a T_1_ energy of ~3.02 eV. With 400 nm visible-light excitation, an almost identical phosphorescence spectrum was obtained, suggesting that T_1_ can indeed be populated by 400 nm photoexcitation even at the single-molecular state in dilute solution (Figure 3c). Since the energy of 400 nm excitation and 408 nm phosphorescence is very close, we propose that T_1_ would be directly populated under visible-light excitation through S_0_→T_1_ absorption [29]. Excitation spectra of the phosphorescence peak in 2-Me THF were examined with increasing solution concentrations. It was observed that the primary peak of the phosphorescence excitation spectrum underwent a red shift, along with the emergence of new excitation peaks in the longer-wavelength range (Figure S23). This phenomenon indicates that as the solution concentration increases, the intermolecular heavy-atom effect becomes more pronounced, which is essential for achieving direct S_0_→T_1_ absorption.

In addition to the 400 nm excitable phosphorescence used to confirm the direct population of T_1_, we conducted photodegradation experiments using 1,3-Diphenylisobenzofuran (**DPBF**) in the presence of the organic afterglow molecules and oxygen (Figure 3d) [35]. The characteristic absorption peaks (415 nm) of **DPBF** in the **PC2Br** solution gradually decreased in intensity with prolonged irradiation time, directly indicating the existence of triplet excited-states upon 400 nm photoexcitation. Control experiments were also conducted to verify the unique S_0_→T_1_ absorption in these heavy-atom molecules (Figure S24).

To gain a theoretical understanding of the direct S_0_→T_1_ absorption, we conducted first-principle time-dependent density functional theory (TD-DFT) investigations on these molecules (Figure S25) [36]. The calculated S_1_ energy levels are considerably close (<0.3 eV) to several T_n_, supporting facile ISC channels for S_1_→T_n_ transitions in accordance with the energy gap law of **PC2Br**. Notably, these SOC values are significantly larger than those of heavy atom-free organic molecules (<0.1 cm^−1^) (Figure 3e) [37]. Therefore, the 295 nm excited S_1_ state can undergo transformation to T_n_ states through these ISC channels, and the T_n_ states can further relax to T_1_ for organic afterglow through the traditional path to populate triplet states. More importantly, these heavy atom-modified molecules exhibit very high SOC values for the S_0_→T_1_ transition, even surpassing those of their S_1_→T_n_ transitions, providing computational evidence for the direct S_0_→T_1_ absorption.

Based on the experimental and theoretical evidence, a two-way organic afterglow mechanism can be proposed (Figure 3f). The first way is traditional, involving four steps: S_0_→S_n_ absorption and internal conversion (IC) between S_n_, S_1_→T_n_ ISC, IC between T_n_, and triplet exciton trapping to form stabilized T_1_ in aggregated structures ($T_{1}^{*}$) leading to the organic afterglow. The second pathway to populate T_1_ for afterglow is direct S_0_→T_1_ absorption, bypassing the traditional ISC (S_1_→T_n_) and IC processes, followed by exciton trapping to form $T_{1}^{*}$ leading to the organic afterglow. However, this way for organic afterglow involves only two excited states, T_1_ and $T_{1}^{*}$, while the radiative and non-radiative decays of other excited states needed in the first pathway are completely avoided. Therefore, the organic afterglow performance is significantly improved and the excitation wavelength is red-shifted compared to the traditional UV-light excitation method.

### 2.3. Crystal Structure Analysis

To gain deeper insights into the exceptional organic afterglow emission exhibited by these compounds, we investigated the molecular packing structures based on their single-crystal configurations [38]. Notably, robust π–π stacking involving two carbazole rings positioned parallel to each other was evident in the single crystals of **PC2Br**, **PC3Br**, **PC27DBr**, and **PC36DBr** (Figure S13). Despite not stacking entirely face-to-face due to the repulsion between the π electrons on each aryl ring, this closely packed configuration fosters efficient electronic intermolecular interactions between molecular orbitals. It is crucial to note that SOC calculation relies on isolated single molecular states, potentially overlooking intermolecular heavy-atom effects that enhance SOC. Indeed, apparent weak interactions between Br and the aromatic moiety of its adjacent molecule were identified through non-bonding covalent interaction (NCI) analysis (Figure S26) [39,40]. Therefore, an even more efficient S_0_→T_1_ transition can be anticipated in the condensed solid states through the intermolecular heavy-atom effect of these brominated molecules.

### 2.4. Lifetime-Resolved Flexible Pattern Encryption

**Figure 4:**
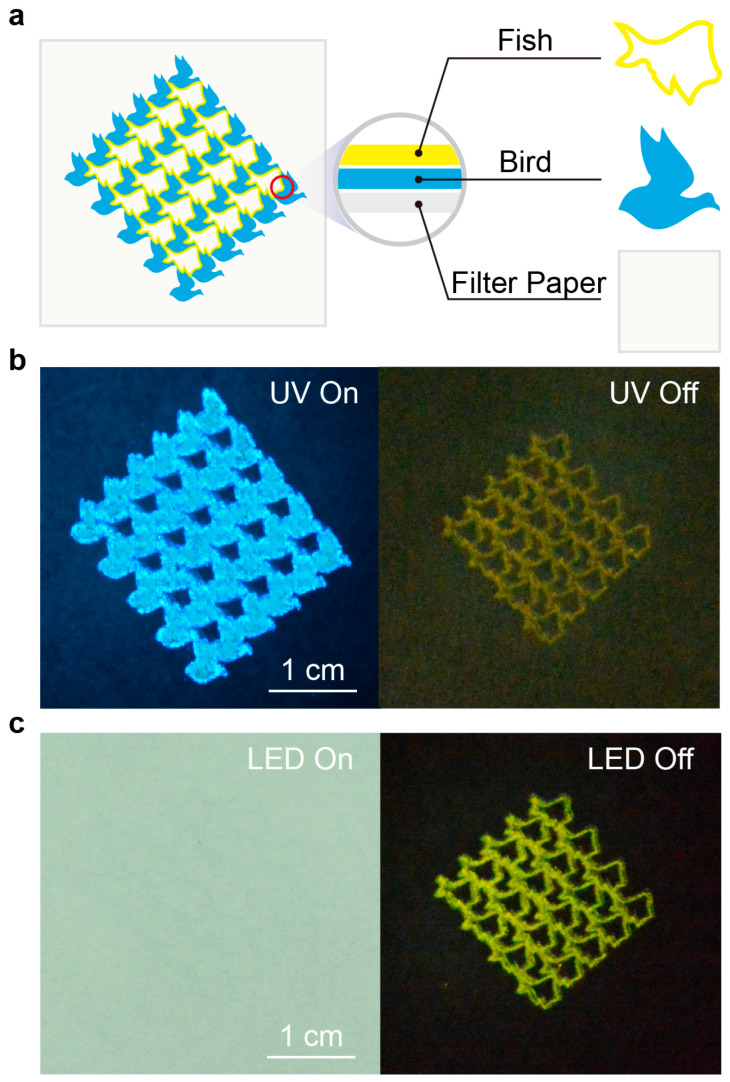
Color-encoded and excitation wavelength-dependent pattern encryption. (**a**) Device structure of the flexible pattern encryption. Photographs of the device under (**b**) 365 nm UV and (**c**) white LED irradiation (**left**), as well as after the removal of the excitation (**right**).

Given the remarkable high-efficiency visible light-excited organic afterglow emission via direct S_0_→T_1_ absorption, we open avenues for lifetime-resolved, color-encoded and excitation wavelength-dependent pattern encryption by using heavy-atom incorporated organic afterglow molecules and commercially available fluorescent compounds on flexible filter paper substrates [29,41]. A fish outline was rendered using the organic afterglow molecule **PC36DBr**, and birds were painted in blue using the fluorescent dye bis[4-(9,9-dimethyl-9,10-dihydroacridine)]phenylsulfone (**DMAC-DPS**) (Figure 4a) [42]. The organic afterglow emitters and fluorescent dye exhibited similar steady-state emission color, ensuring effective encryption of the fish pattern, and only revealing the blue bird under a 365 nm UV lamp. Once the excitation lamp was turned off, the fish outline emerged in yellow afterglow emission, visible to the naked eye (Figure 4b). Furthermore, as **DMAC-DPS** and **PC36DBr** fluorescence cannot be excited by a white LED, the fish pattern remained effectively encrypted, with no discernible pattern under white LED illumination. Upon turning off the white LED, the fish outline became vividly visible in a brighter yellow afterglow emission (Figure 4c).

## 3. Materials and Methods

### 3.1. Synthesis and Characterization

Chemicals and solvents obtained from Aldrich or Acros, meeting analytical grade standards, were utilized without further purification. Unless otherwise specified, reactions were conducted under a dry argon atmosphere employing standard Schlenk techniques. ^1^H and ^13^C nuclear magnetic resonance (NMR) spectra were acquired using a Bruker (Billerica, MA, USA) Ultra Shield Plus 400 MHz instrument, with CDCl_3_ or DMSO as the solvent and tetramethylsilane (TMS) serving as the internal standard. Chemical shifts (δ) were reported in ppm and Hz. Splitting patterns were assigned as follows: s (singlet), d (doublet), and m (multiplet). LC-MS was performed on an Agilent (Santa Clara, CA, USA) 6230 Accurate-Mass TOF LC/MS using a mixed solvent of 50 vol% water and 50 vol% methanol as the eluent.

**2-Bromo-9-phenyl-9H-carbazole (PC2Br):** In a 100 mL round-bottom flask equipped with a stir bar, 2-bromo-9H-carbazole (1.00 g, 4.06 mmol), copper iodide (0.85 g, 4.47 mmol), potassium carbonate (1.12 g, 8.13 mmol), iodobenzene (0.68 mL, 1.24 g, 6.09 mmol), and 50 mL dry N, N-dimethylformamide (DMF) were combined. The mixture, under nitrogen protection, was stirred and allowed to react for 24 h at 180 °C. Following cooling to room temperature, the DMF solvent was removed by vacuum distillation. The resulting solid was dissolved in 120 mL dichloromethane (DCM) and washed with brine (60 mL). Subsequently, the mixture was subjected to three DCM extractions. The organic phase was collected and dried over MgSO_4_. After the solvent removal under reduced pressure, the crude product was purified by column chromatography (using petroleum ether: DCM = 10:1 as the eluent) and recrystallized from DCM/hexane three times to obtain colorless crystals. Yield: 0.86 g (65%). ^1^H NMR (400 MHz, CDCl_3_): δ = 8.14 (d, 1H), 8.02 (d, 1H), 7.65 (m, 2H), 7.59–7.50 (m, 4H), 7.49–7.38 (m, 3H), 7.36–7.30 (m, 1H). ^13^C NMR (100 MHz, CDCl_3_): δ = 141.73, 141.13, 137.09, 130.10, 127.95, 127.16, 126.39, 123.10, 122.76, 122.30, 121.50, 120.42, 120.31, 119.55, 112.83, 110.00. LC-MS (methanol–water 50:50 (*v*/*v*)): *m*/*z* calcd. for C_18_H_12_NBr [M+H]^+^: 322.02; found: 321.19.

**3-Bromo-9-phenyl-9H-carbazole (PC3Br):** Following a procedure similar to the synthesis of **PC2Br**, **PC3Br** was obtained using 3-bromo-9H-carbazole (1.00 g, 4.06 mmol). The yield was 0.93 g of colorless crystals (71%). ^1^H NMR (400 MHz, DMSO): δ = 8.51 (d, 1H), 8.35–8.28 (m, 1H), 7.68 (m, 2H), 7.61 (m, 2H), 7.59–7.53 (m, 2H), 7.47 (m, 1H), 7.39–7.35 (m, 1H), 7.31 (m, 2H). ^13^C NMR (100 MHz, DMSO): δ = 140.99, 139.38, 136.87, 130.73, 129.09, 128.44, 127.55, 127.17, 125.15, 123.59, 122.14, 121.58, 120.91, 112.53, 112.07, 110.28. LC-MS (methanol–water 50:50 (*v*/*v*)): *m*/*z* calcd. for C_18_H_12_NBr [M + H]^+^: 322.02; found: 322.62.

**2,7-Dibromo-9-phenyl-9H-carbazole (PC27DBr):** Following the procedure used for the synthesis of **PC2Br** and **PC3Br**, **PC27DBr** was prepared using 2,7-dibromo-9H-carbazole (1.32 g, 4.06 mmol). The yield was 1.37 g of colorless crystals (84%). ^1^H NMR (400 MHz, CDCl_3_): δ = 7.98–7.96 (d, 1H), 7.68–7.65 (t, 1H), 7.59–7.49 (m, 2H), 7.44–7.41 (m, 1H). ^13^C NMR (100 MHz, CDCl_3_): δ = 141.91, 136.44, 130.29, 128.40, 127.14, 123.62, 121.68, 121.49, 119.99, 113.06, 77.35, 77.03, 76.72. LC-MS (methanol–water 50:50 (*v*/*v*)): *m*/*z* calcd. for C_18_H_11_NBr_2_ [M + H]^+^: 401.93; found: 401.14.

**3,6-Dibromo-9-phenyl-9H-carbazole (PC36DBr):** Similar to the synthesis of **PC2Br**, **PC3Br**, and **PC27DBr**, **PC36DBr** was prepared using 3,6-dibromo-9H-carbazole (1.32 g, 4.06 mmol). The yield was 1.47 g of colorless crystals (90%). ^1^H NMR (400 MHz, DMSO): δ = 8.59 (d, 1H), 7.72–7.66 (m, 1H), 7.63–7.54 (m, 3H), 7.32 (d, 1H). ^13^C NMR (100 MHz, DMSO): δ = 139.76, 136.45, 130.79, 129.94, 128.72, 127.17, 124.16, 124.03, 112.91, 112.34. LC-MS (methanol–water 50:50 (*v*/*v*)): *m*/*z* calcd. for C_18_H_11_NBr_2_ [M + H]^+^: 401.93; found: 401.03.

### 3.2. Single-Crystal X-ray Analysis

Single crystals were obtained through the slow evaporation of combined solutions of combined DCM and hexane at room temperature. X-ray crystallography analysis was performed using a Bruker Smart Apex CCD area detector diffractometer with graphite-monochromated Mo-Kα radiation (λ = 0.71073 Å) at 293 K. The cell parameters were determined using SMART software (Version 5.625) and refined with SAINT on all observed reflections. Structures were solved via direct methods using the SHELX-97 program package (Version 97-2). Non-hydrogen atoms were located using alternating difference Fourier syntheses and least-squared refinement cycles, with the final cycles involving anisotropic refinement. Hydrogen atoms were positioned in calculated positions and refined as riding atoms with a uniform value of *U*_iso_. The crystal structure was analyzed using Diamond 3.2 software. The crystallographic parameters for **PC2Br**, **PC3Br**, **PC27DBr**, and **PC36DBr** are summarized in Table S1. The CCDC reference numbers are Nos. 1973898, 1973897, 1973900, and 1973899, respectively.

### 3.3. Photophysical Property Investigations

UV-Vis spectra were recorded with a SHIMADZU (Tokyo, Japan) UV-3600 UV-VIS-NIR spectrophotometer. SSPPL and phosphorescence spectra, along with quantum yields, were measured using an Edinburgh FLSP980 fluorescence spectrophotometer. For steady-state photoluminescence and quantum yield measurements, we utilized a xenon arc lamp (Xe900), known for its excellent steady-state excitation capabilities. When capturing phosphorescence spectra, organic afterglow spectra, organic afterglow decay, and time-resolved excitation spectra, we employed a microsecond flash lamp (uF900) in conjunction with a gating technique. The uF900 flash lamp emits short (typically a few microseconds) and high-irradiance optical pulses suitable for measurements ranging from microseconds to seconds. Additionally, LED flash lamps were utilized to provide subnanosecond optical pulses across the UV-to-visible spectral range for fluorescence decay measurements. To ensure accurate steady-state spectra measurements, we adjusted the excitation wavelength from 400 nm to 395 nm and incorporated a 400 nm filter to mitigate the influence of stray light. The lifetimes (*τ*) of luminescence were determined by fitting the decay curve with a multi-exponential decay function.

(1) $$I\left(t\right)=\sum_{i}A_{i}e^{-\frac{t}{\tau }}$$

where *A_i_* and *τ_i_* represent the amplitudes and lifetimes of the individual components for multi-exponential decay profiles, respectively.

The quantum yields (*φ*) of the emission were the absolute ones performed by photon counting from the excitation source into an integration sphere with the ratio of photons emitted, as described in the following equation:

(2) $$\varphi =\frac{N^{em}}{N^{abs}}$$

In this equation, *N^em^* is the number of emitted photons and *N^abs^* is the number of absorbed photons. The organic afterglow efficiency is determined through peak differentiation-imitating analysis from the steady-state PL spectra.

Phosphorescence excitation–emission mapping was performed on a Hitachi (Tokyo, Japan) Fluorescence Spectrophotometer F-4700. The photographs were recorded by a Nikon D90 camera.

### 3.4. Probing the Formation of Triplet Excited States

To validate the generation of triplet excited states upon 400 nm excitation, experiments were conducted using well-dissolved **PC2Br** molecules (20 μM) in THF containing **DPBF** (50 μM) and dissolved molecular oxygen. A control experiment with only **DPBF** in THF (50 μM) was also performed, revealing negligible changes in the absorption spectra of **DPBF** after excitation at 400 nm for 15 min.

### 3.5. TD-DFT Calculations

TD-DFT calculations were conducted at the B3LYP/6-31G(d) level using the Gaussian 09 package. The S_0_ geometry was fully optimized with B3LYP/6-31G(d), and the optimized stationary point underwent harmonic vibration frequency analysis to ensure the identification of real local minima. The excitation energies in the S_n_ and T_n_ states were obtained using the TD-DFT method based on the optimized molecular structure at S_0_. SOC matrix elements between the singlet and triplet excited states were calculated using quadratic response function methods with the Dalton program at the optimized geometry of S_1_, utilizing the B3LYP functional and 6-31G(d) basis set. Additionally, the SOC between the ground state and the T_1_ was calculated using the same method but based on the optimized ground state geometry.

The intermolecular interactions between the heavy atom of Br and the π-conjugated moiety of the nearby molecule were analyzed using NCI analysis using Multiwfn version 3.3 software based on typical molecular packing structures in single crystals with B3LYP/6-31G(d). NCI isosurface plots were performed with color scaling in which the dark blue color represents attractive interactions, while the dark red color represents repulsive interactions. The plotted isosurfaces were demonstrated with RDG of 0.5 and −0.05 < *sign*(*λ*_2_)*ρ* < 0.05, where *sign*(*λ*_2_) denotes the sign of the second-largest Hessian eigenvalue, and *ρ* represents the electron density.

### 3.6. Flexible Pattern Encryption Application

Time-resolved, color-encoded and excitation wavelength-dependent encrypted images were fabricated as follows. On a filter paper substrate, the fish outline was first rendered using the organic afterglow molecule **PC36DBr** in Aloe vera gel (c.a. 40 mg/mL) by silk-screen printing. In the second step, the birds were painted in a thin film of a blue dye using the **DMAC-DPS** dispersed in Aloe vera gel (c.a. 40 mg/mL) using the same technology.

## 4. Conclusions

In summary, we have successfully developed a series of organic afterglow molecules capable of the direct population of triplet excited states through direct S_0_→T_1_ absorption. This achievement was realized by incorporating both intra- and intermolecular heavy-atom effects synergistically in aromatic organic crystals. The facilitated S_0_→T_1_ transition, validated through a combination of experimental and theoretical investigations, imparts unique features to organic afterglow, such as significantly improved organic afterglow efficiency, visible-light excitability, and a prolonged emission lifetime under ambient conditions. Furthermore, based on the strong and efficient organic afterglow, we successfully fabricated a flexible pattern-encryption device that is lifetime-resolved, color-encoded, and excitation wavelength-dependent. These findings provide new insights into the direct population of triplet excited states in metal-free organic optoelectronic molecules, and contribute fundamentally to our understanding of the organic afterglow mechanism and the molecular design of highly efficient visible-light excitable organic afterglow materials.

## Figures and Tables

**Table 1:** Photophysical properties of the molecules in the crystal at room temperature.

Comp.	Fluorescence		Organic Afterglow ^1^			Organic Afterglow ^2^		
	λ (nm)	τ (ns)	λ (nm)	τ (ms)	φ (%)	λ (nm)	τ (ms)	φ (%)
**PhCz**	380	11.2	548	240	1.50	548	260	1.80
**PC2Br**	380	4.3	526	63	0.37	526	157	5.81
**PC3Br**	390	2.1	536	60	0.93	536	70	4.38
**PC27DBr**	397	2.4	572	50	0.56	572	88	3.36
**PC36DBr**	404	3.3	571	48	0.71	571	66	2.26

^1^ Excited at 295 nm; ^2^ excited at 400 nm.

## Data Availability

Data are contained within the article and Supplementary Materials.

## Supplementary Materials

The following supporting information can be downloaded at: https://www.mdpi.com/article/10.3390/molecules29051014/s1. Figure S1: ^1^H NMR spectrum of **PC2Br**; Figure S2: ^13^C NMR spectrum of **PC2Br**; Figure S3: ^1^H NMR spectrum of **PC3Br**; Figure S4: ^13^C NMR spectrum of **PC3Br**; Figure S5: ^1^H NMR spectrum of **PC27DBr**; Figure S6: ^13^C NMR spectrum of **PC27DBr**; Figure S7: ^1^H NMR spectrum of **PC36DBr**; Figure S8: ^13^C NMR spectrum of **PC36DBr**; Figure S9: Liquid chromatographs of **PC2Br** after the recrystallization; Figure S10: Liquid chromatographs of **PC3Br** after the recrystallization; Figure S11: Liquid chromatographs of **PC27DBr** after the recrystallization; Figure S12: Liquid chromatographs of **PC36DBr** after the recrystallization; Figure S13: Single-crystal structures of **PC2Br, PC3Br, PC27DBr**, and **PC36DBr**; Table S1: Structure data of **PC2Br**, **PC3Br**, **PC27DBr**, and **PC36DBr** single crystals; Figure S14: UV-Vis absorption spectra and SSPL spectra of **PC2Br**, **PC3Br**, **PC27DBr**, and **PC36DBr** in (a~b) CH_2_Cl_2_ dilute solution (~10^−5^ mol L^−1^) and (c~d) thin films, excited by 295 nm at room temperature; Figure S15: Fluorescence decay curves of (a) **PC2Br**, (b) **PC3Br**, (c) **PC27DBr**, and (d) **PC36DBr** in dilute CH_2_Cl_2_ solutions (10^−5^ mol L^−1^) excited by 295 nm irradiation at room temperature; Figure S16: Fluorescence decay curves of (a) **PC2Br**, (b) **PC3Br**, (c) **PC27DBr**, and (d) **PC36DBr** in thin films excited by 295 nm irradiation at room temperature; Figure S17: Fluorescence decay curves of (a) **PC2Br**, (b) **PC3Br**, (c) **PC27DBr**, and (d) **PC36DBr** crystals excited by 295 nm irradiation at room temperature; Figure S18: Steady-state and organic afterglow emission spectra of **PC2Br**, **PC3Br**, **PC27DBr**, and **PC36DBr** crystals excited at 395 nm with a 400 nm filter at room temperature; Figure S19: organic afterglow decay curves of (a) **PC2Br**, (b) **PC3Br**, (c) **PC27DBr**, and (d) **PC36DBr** crystals excited at 295 nm and 400 nm at room temperature; Figure S20: Excitation spectra for organic afterglow peaks of (a) **PC2Br**, (b) **PC3Br,** (c) **PC27DBr**, and (d) **PC36DBr** crystals at room temperature; Figure S21: Steady-state emission spectrum of white LED at room temperature; Figure S22: UV-light (295 nm, black line) and visible-light (400 nm, red line) excited phosphorescence spectra of (a) **PC2Br**, (b) **PC3Br**, (c) **PC27DBr**, and (d) **PC36DBr** in 2-methyltetrahydrofuran dilute (~10^−5^ mol L^−1^) solution at 77 K with a delay time of 10 ms; Figure S23: Phosphorescence excitation spectra of **PC2Br** in 2-methyltetrahydrofuran solution with increasing concentration at 77 K, recorded with a delay time of 10 ms; Figure S24: Absorption spectra of **DPBF** (50 μM) in THF under 400 nm irradiation for 15 min at room temperature; Figure S25: Schematic diagrams showing the TD-DFT-calculated energy levels at singlet (S_n_) and triplet (T_n_) states and spin-orbit coupling (SOC) values for S_1_→T_n_ and S_0_→T_n_ of (a) **PC2Br**, (b) **PC3Br,** (c) **PC27DBr**, and (d) **PC36DBr.** Note that only the most possible T_n_ with singlet-triplet splitting (Δ*E*_ST_) lower than 0.3 eV are illustrated; Figure S26: Analysis of intermolecular heavy-atom effect. Plots of reduced density gradient (RDG) versus *sign*(*λ*_2_)*ρ* with RDG isosurface of the selected dimers in (a) **PC2Br**, (b) **PC3Br**, (c) **PC27DBr**, and (d) **PC36DBr** single crystals. Intermolecular heavy-atom interactions are highlighted in red circles.
