# Supplementary material for: Direct Population of Triplet States for Efficient Organic Afterglow through the Intra/Intermolecular Heavy-Atom Effect
Source: Molecules. 2024 Feb 26;29(5):1014. doi: 10.3390/molecules29051014 (PMC10934877; doi:10.3390/molecules29051014)
Supplement: Supplementary file 1 [file molecules-29-01014-s001.zip › molecules-2871059-supplementary.pdf]

Electronic Supporting Information (ESI)

Direct Population of Triplet States for Efficient Organic Afterglow through the Intra/Intermolecular Heavy-Atom Effect

Jie Yuan [1,2], Yongrong Wang [2], Binbin Zhou [1], Wenjing Xie [1], Botao Zheng [2], Jingyu Zhang [2], Ping Li [2], Tian Yu [1], Yuanyuan Qi [2], Ye Tao [2,]* and Runfeng Chen [2,]*

[1] Engineering Technology Training Center, Nanjing Vocational University of Industry Technology, 1 Yangshan North Road, Nanjing 210023, China; shuipingyuanjie@niit.edu.cn (J.Y.); 2020101101@niit.edu.cn (B.Z.); WhitneyXie8894@outlook.com (W.X.); 2015100812@niit.edu.cn (T.Y.)

[2] State Key Laboratory of Organic Electronics and Information Displays & Institute of Advanced Materials (IAM), Nanjing University of Posts & Telecommunications, 9 Wenyuan Road, Nanjing 210023, China; iamyrwang@163.com (Y.W.); 15934162709@163.com (B.Z.); sxxyzjy@126.com (J.Z.); iampingli@njupt.edu.cn (P.L.); iamyyqi@njupt.edu.cn (Y.Q.)

* Correspondence: iamytao@njupt.edu.cn (Y.T.); iamrfchen@njupt.edu.cn (R.C.)

Table of Contents

Synthesis and characterization..2

Single crystal X-ray analysis 6

Photophysical property investigations 9

Probing the formation of triplet excited states 18

Time-dependent density functional theory (TD-DFT) calculations 19

Synthesis and characterization

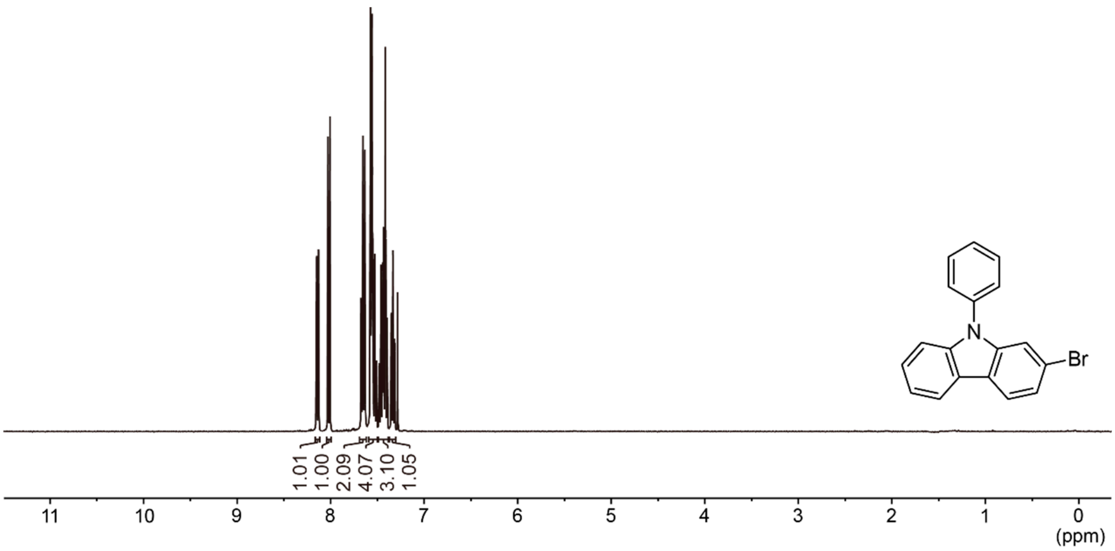

Figure S1. ^{1}H NMR spectrum of **PC2Br**.

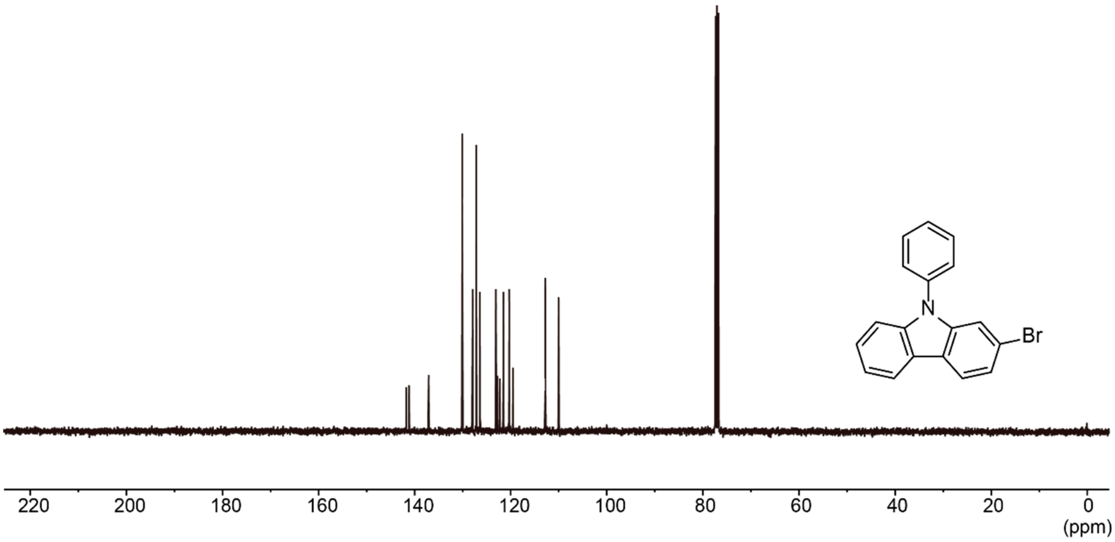

Figure S2. ^{13}C NMR spectrum of **PC2Br**.

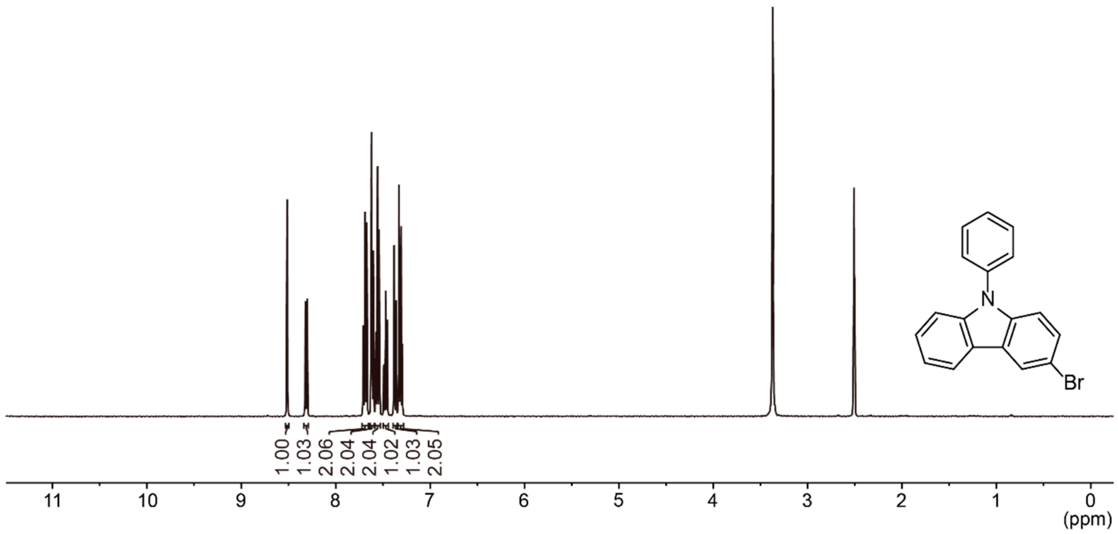

Figure S3. ^{1}H NMR spectrum of **PC3Br**.

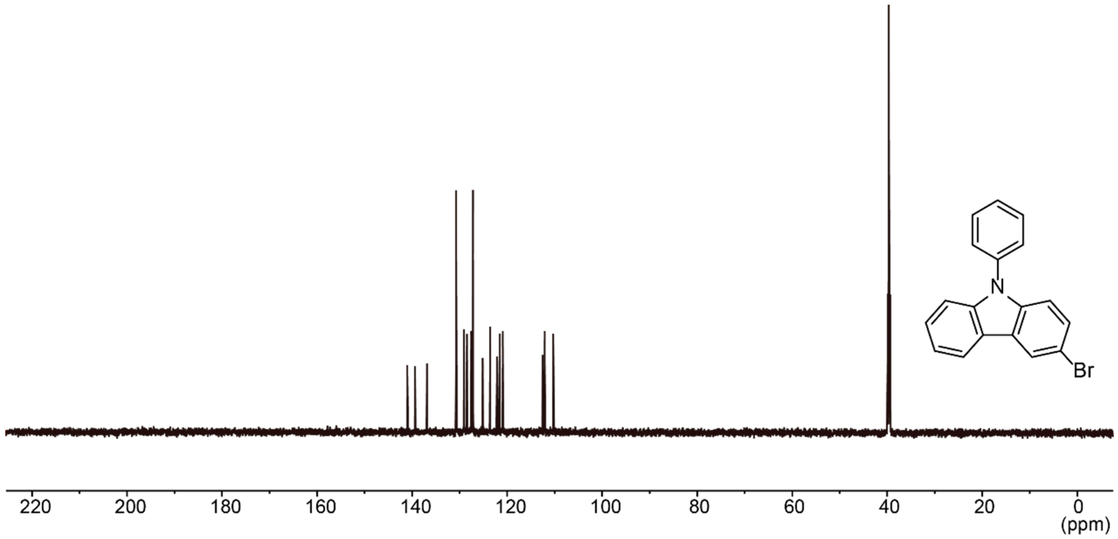

Figure S4. ^{13}C NMR spectrum of **PC3Br**.

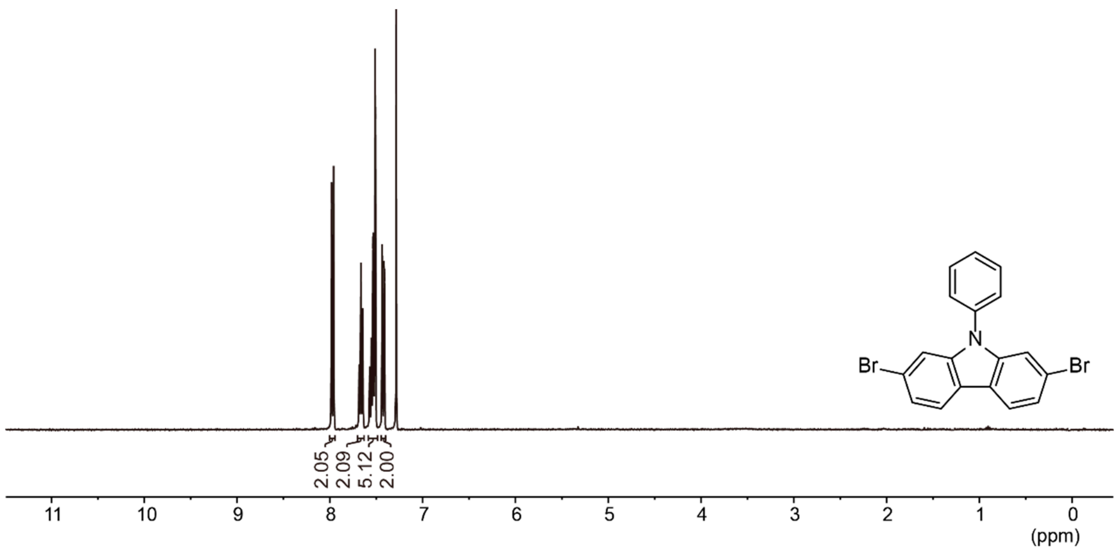

Figure S5. ^{1}H NMR spectrum of **PC27DBr**.

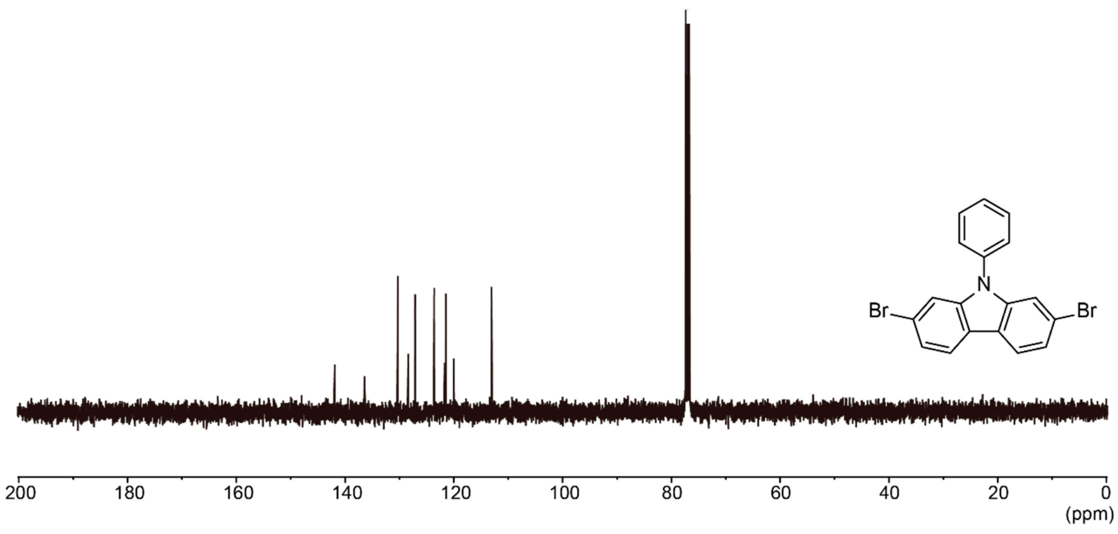

Figure S6. ^{13}C NMR spectrum of **PC27DBr**.

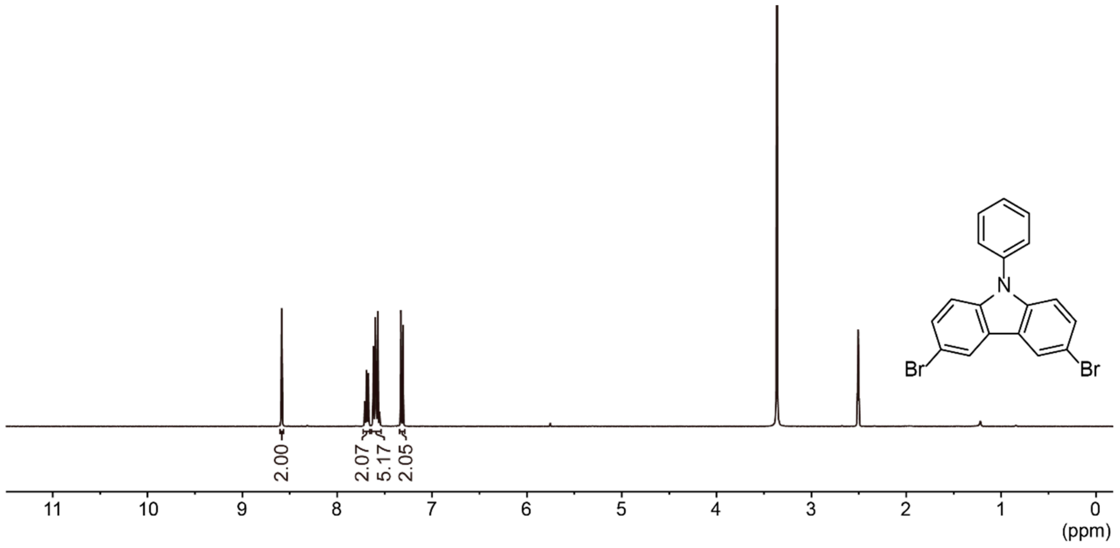

Figure S7. ^{1}H NMR spectrum of **PC36DBr**.

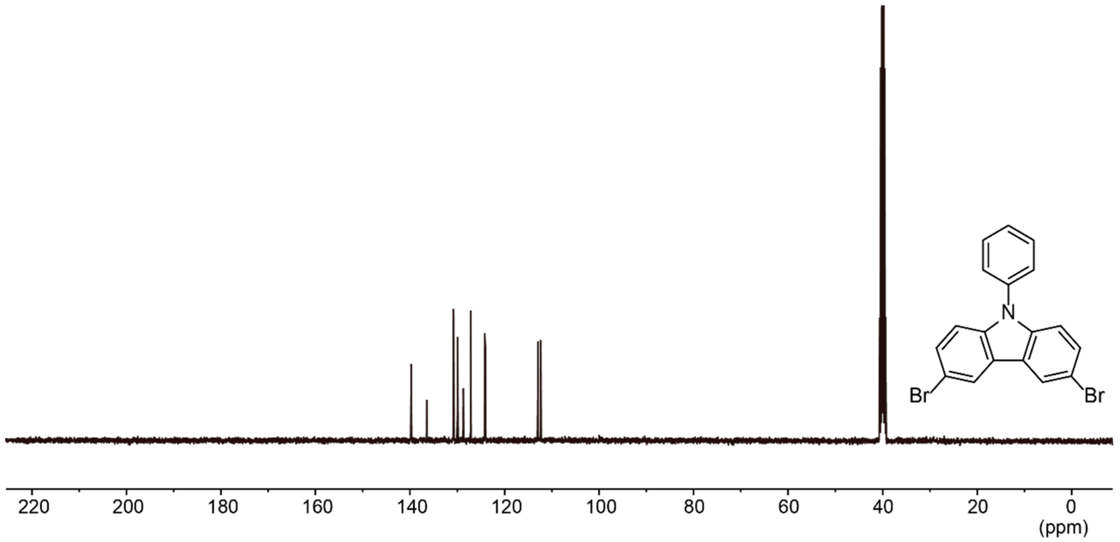

Figure S8. ^{13}C NMR spectrum of **PC36DBr.**

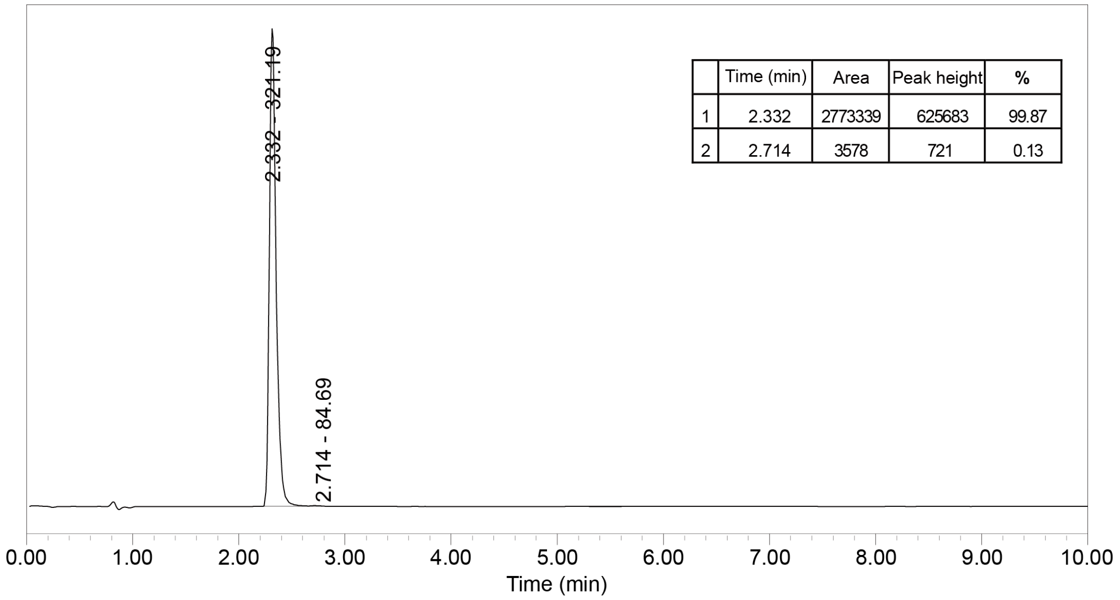

	Time (min)	Area	Peak height	%
1	2.332	2773339	625683	99.87
2	2.714	3578	721	0.13

Figure S9. Liquid chromatographs of **PC2Br** after the recrystallization.

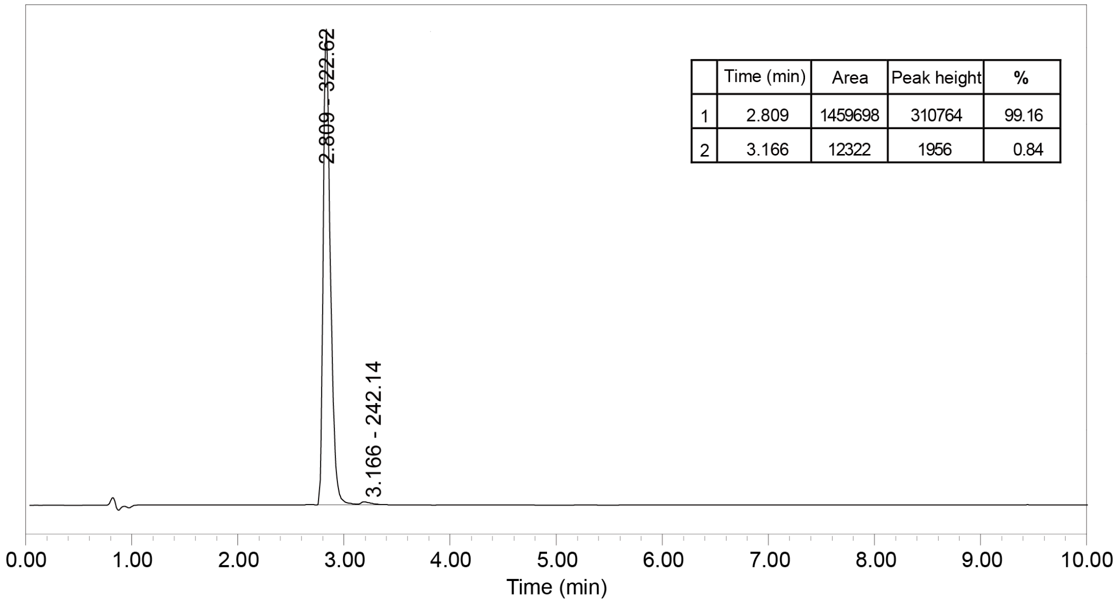

	Time (min)	Area	Peak height	%
1	2.809	1459698	310764	99.16
2	3.166	12322	1956	0.84

Figure S10. Liquid chromatographs of **PC3Br** after the recrystallization.

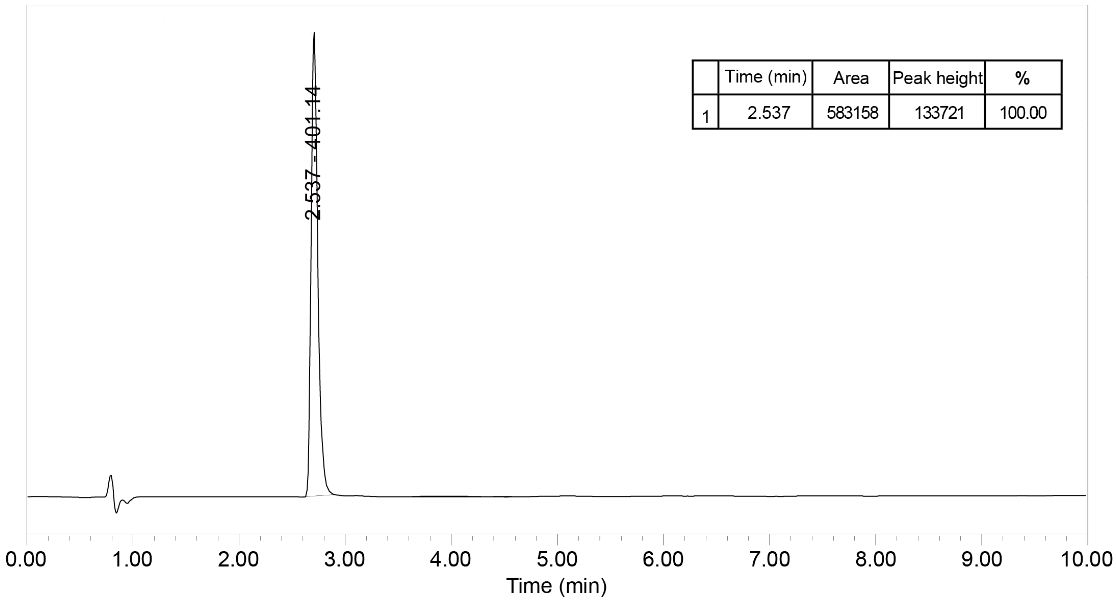

	Time (min)	Area	Peak height	%
1	2.537	583158	133721	100.00

Figure S11. Liquid chromatographs of **PC27DBr** after the recrystallization.

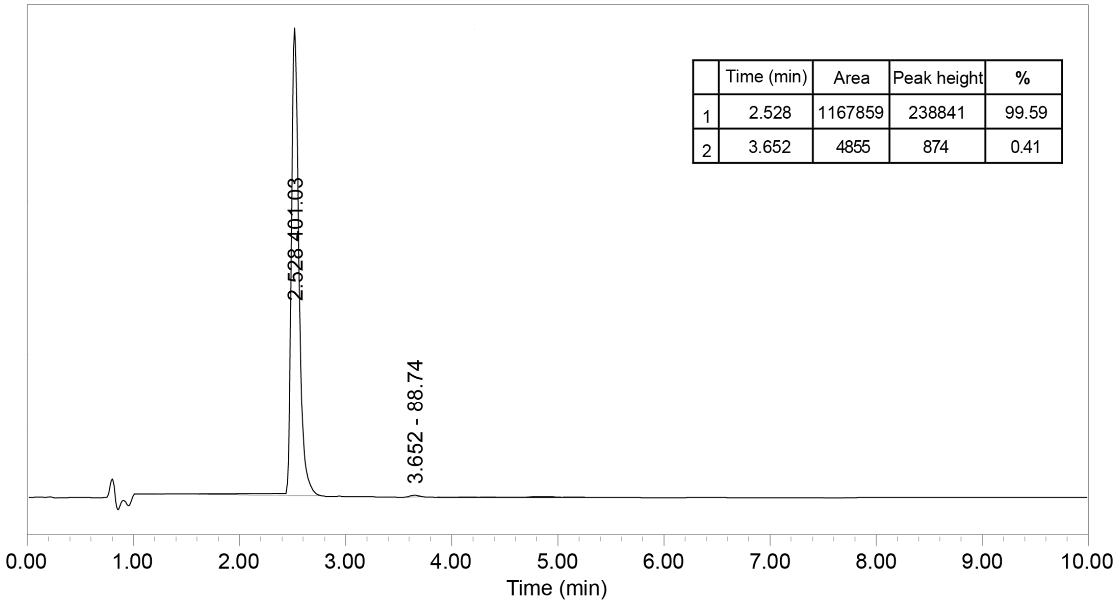

	Time (min)	Area	Peak height	%
1	2.528	1167859	238841	99.59
2	3.652	4855	874	0.41

Figure S12. Liquid chromatographs of **PC36DBr** after the recrystallization.

Single crystal X-ray analysis

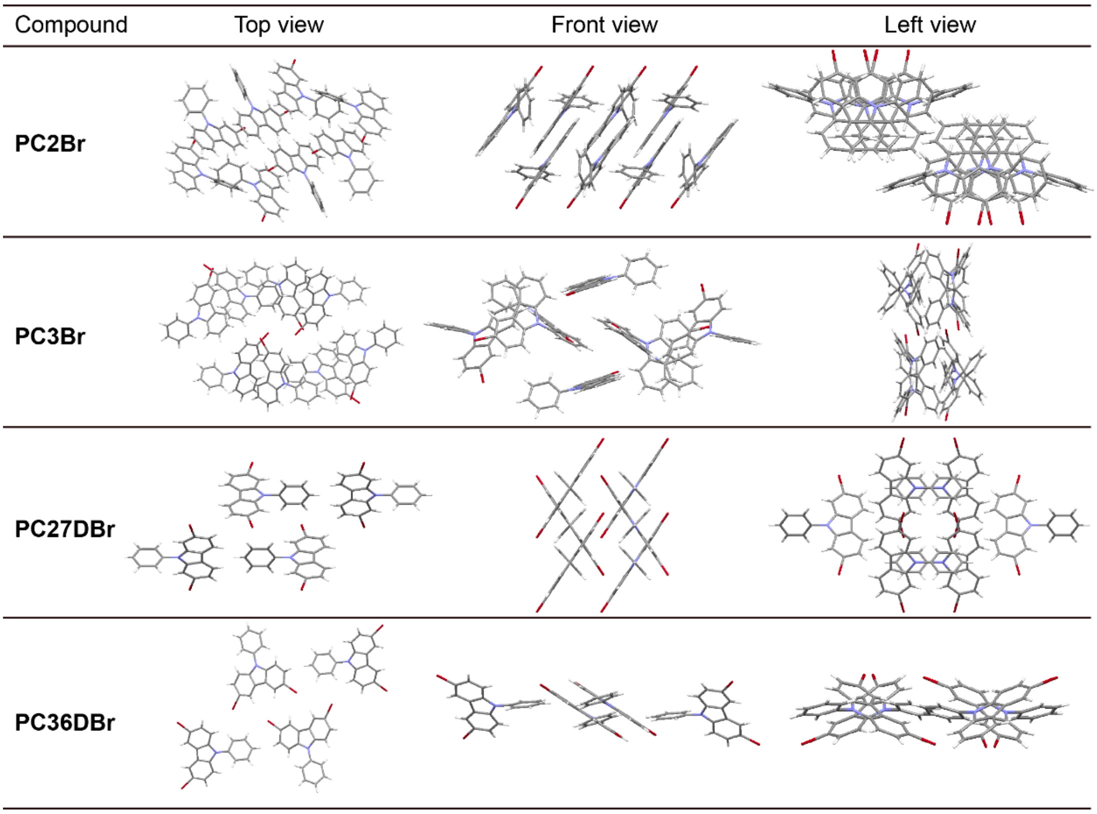

Figure S13. Single-crystal structures of **PC2Br, PC3Br, PC27DBr** and **PC36DBr**.

Table S1. Structure data of **PC2Br, PC3Br, PC27DBr** and **PC36DBr** single crystals.

Compound	**PC2Br**	**PC3Br**	**PC27DBr**	**PC36DBr**
Empirical formula	$C_{18}H_{12}NBr$	$C_{18}H_{12}NBr$	$C_{18}H_{11}NBr_2$	$C_{18}H_{11}NBr_2$
Formula weight (g mol^{-1})	322.2	322.2	401.1	401.1
Crystal color	colorless	colorless	colorless	colorless
Wavelength (Å)	0.71073	0.71073	0.71073	0.71073
Space Group	$P\ 2_1/c$	$P\ 2_1/c$	P bcn	$P\ 2_1/c$
a (Å)	10.438(2)	8.5846(15)	6.5819(8)	4.1487(7)
b (Å)	15.526(4)	17.271(3)	20.537(2)	16.589(3)
c (Å)	17.317(4)	19.703(3)	11.4331(13)	21.702(4)
$\boldsymbol{\alpha}$ (deg)	90	90	90	90
$\boldsymbol{\beta}$ (deg)	92.220(7)	98.009(5)	90	94.621(5)
$\boldsymbol{\gamma}$ (deg)	90	90	90	90
V ($Å^3$)	2804.1(11)	2892.8(8)	1545.4(3)	1488.7(4)
Z	8	8	4	4
Density (g cm^{-3})	1.526	1.480	1.724	1.790
μ (mm^{-1})	2.920	2.830	5.237	5.436
F(000)	1296	1296	784	784
h_{max}, k_{max}, l_{max}	14, 15, 23	11, 16, 26	11, 32, 19	6, 18, 32
$Theta_{max}$	29.858	28.261	37.116	33.059

Photophysical property investigations

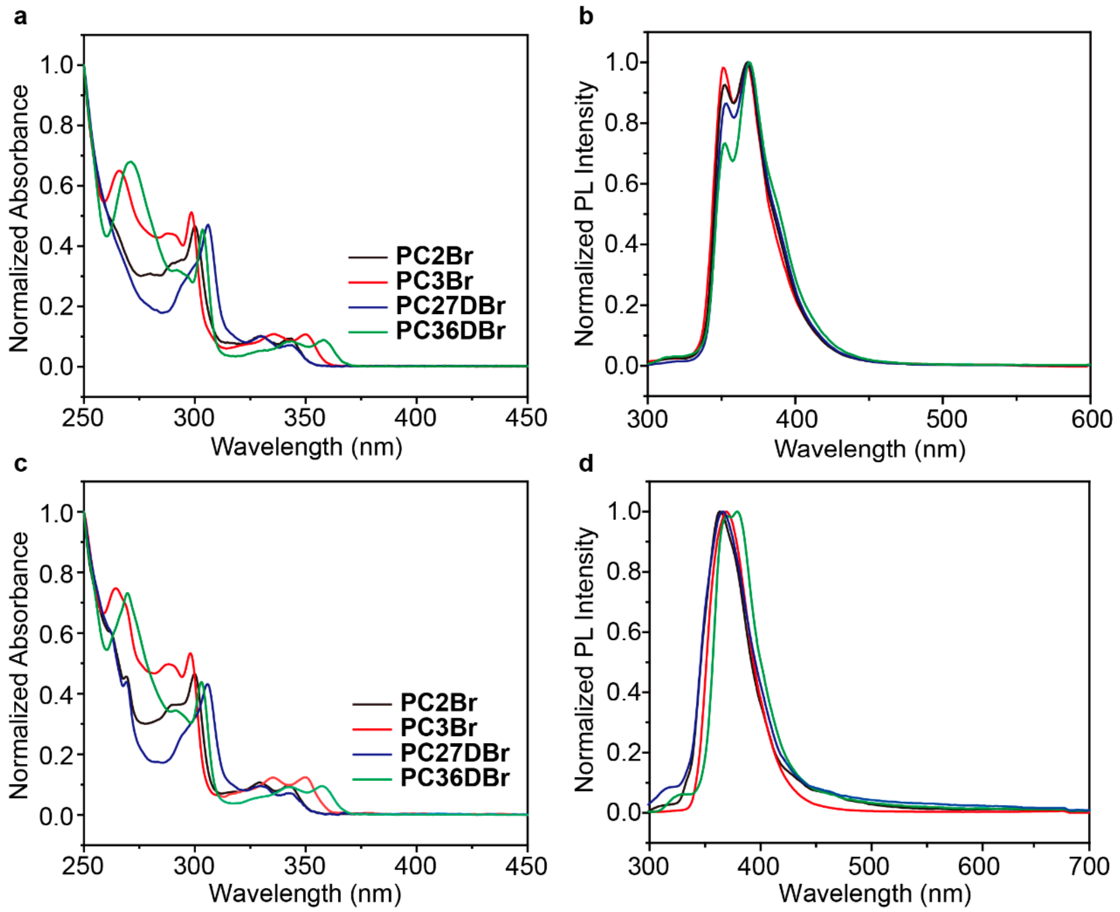

Figure S14. UV-Vis absorption spectra and SSPL spectra of **PC2Br, PC3Br, PC27DBr** and **PC36DBr** in (a~b) CH_2Cl_2 dilute solution (~10^{-5} mol L^{-1}) and (c~d) thin films, excited by 295 nm at room temperature.

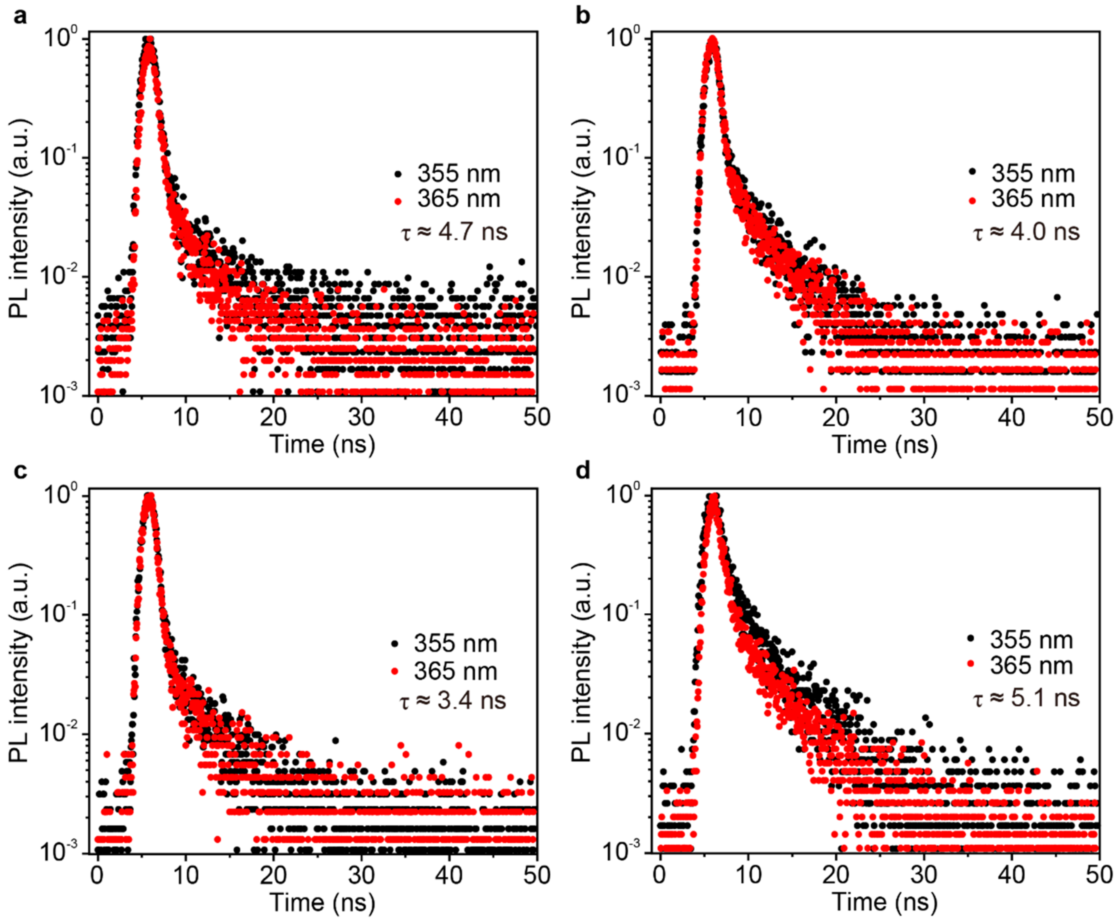

Figure S15. Fluorescence decay curves of (a) **PC2Br,** (b) **PC3Br,** (c) **PC27DBr** and (d) **PC36DBr** in dilute CH_2Cl_2 solutions (10^{-5} mol L^{-1}) excited by 295 nm irradiation at room temperature.

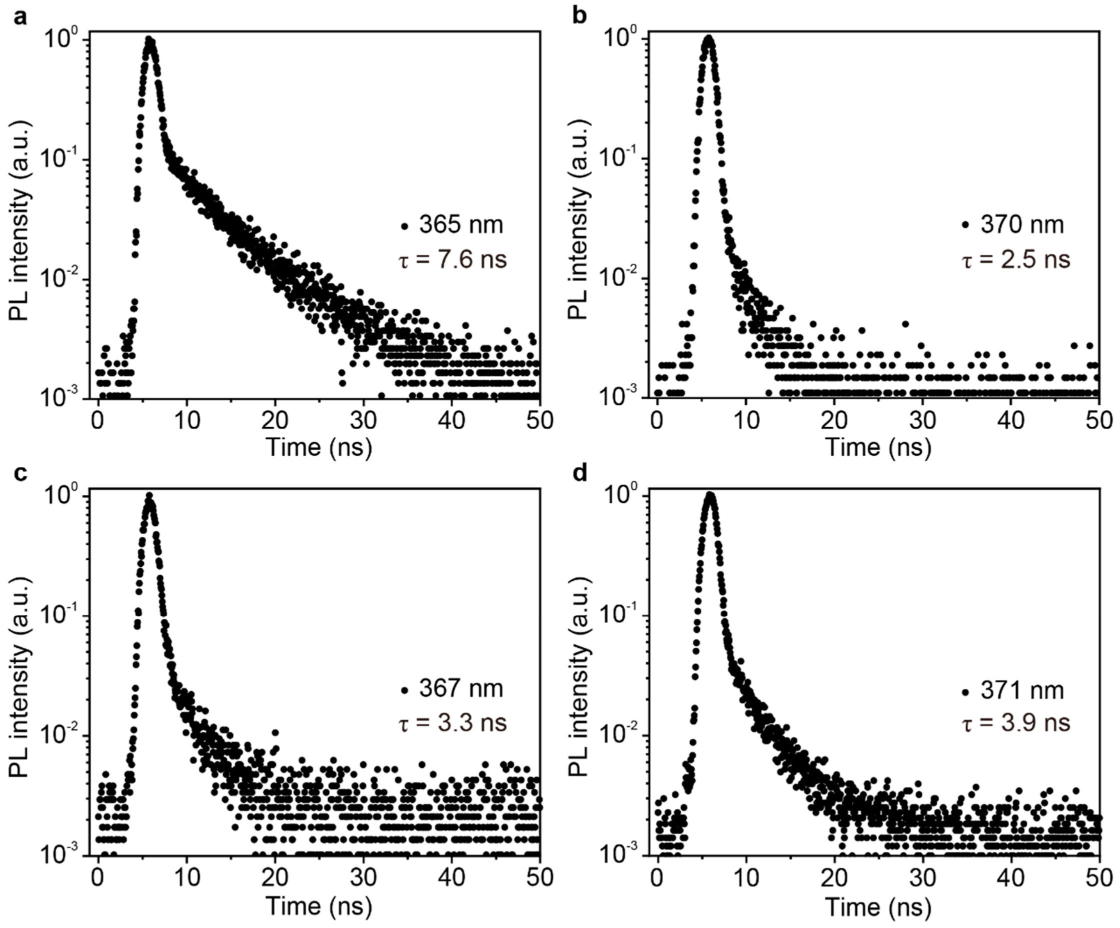

Figure S16. Fluorescence decay curves of (a) **PC2Br,** (b) **PC3Br,** (c) **PC27DBr** and (d) **PC36DBr** in thin films excited by 295 nm irradiation at room temperature.

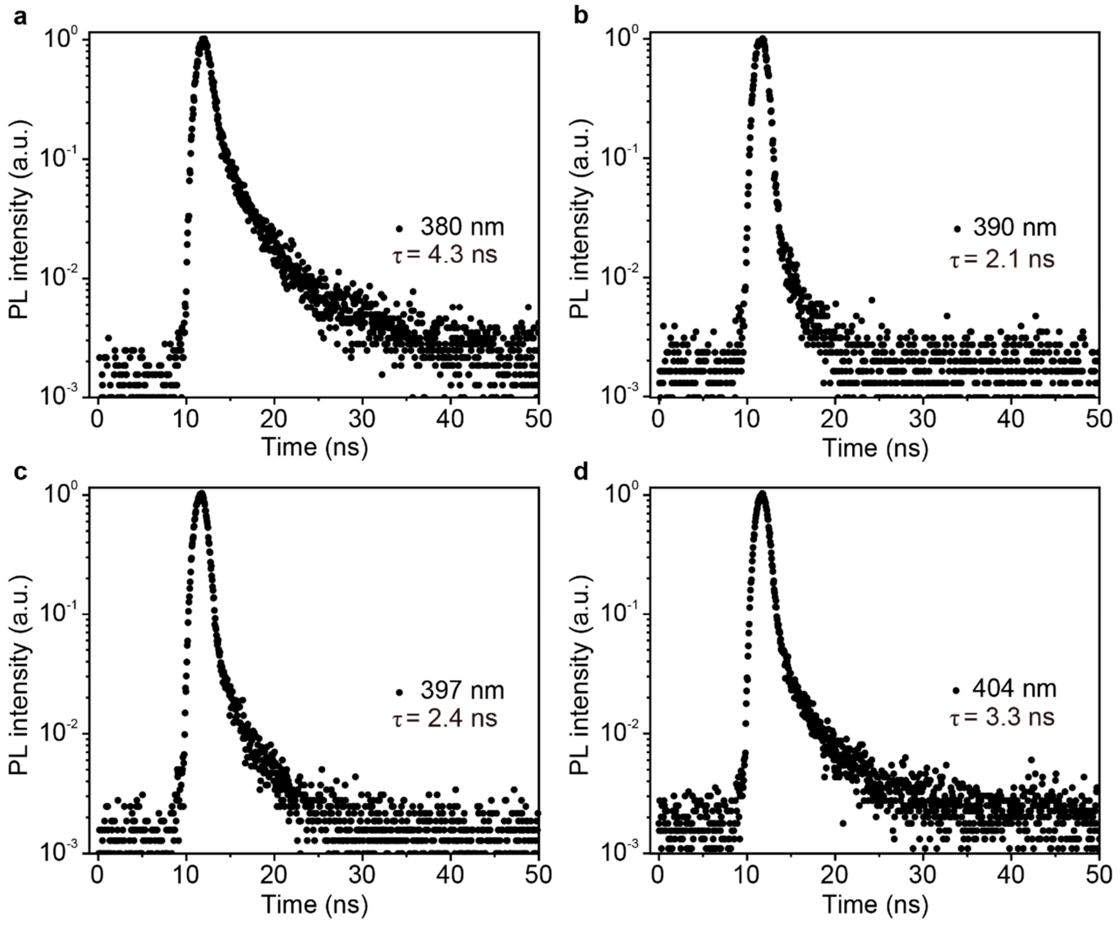

Figure S17. Fluorescence decay curves of (a) **PC2Br,** (b) **PC3Br,** (c) **PC27DBr** and (d) **PC36DBr** crystals excited by 295 nm irradiation at room temperature.

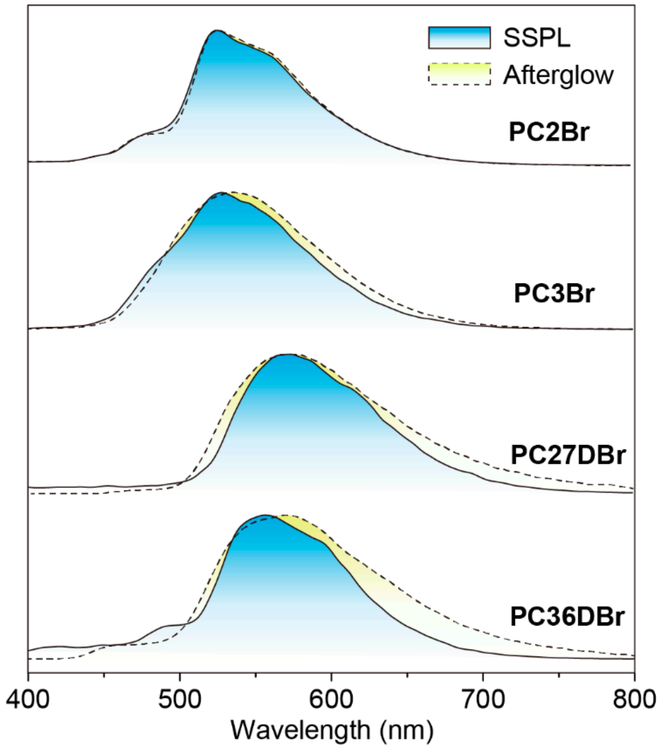

Figure S18. Steady-state and organic afterglow emission spectra of **PC2Br, PC3Br, PC27DBr** and **PC36DBr** crystals excited at 395 nm with a 400 nm filter at room temperature.

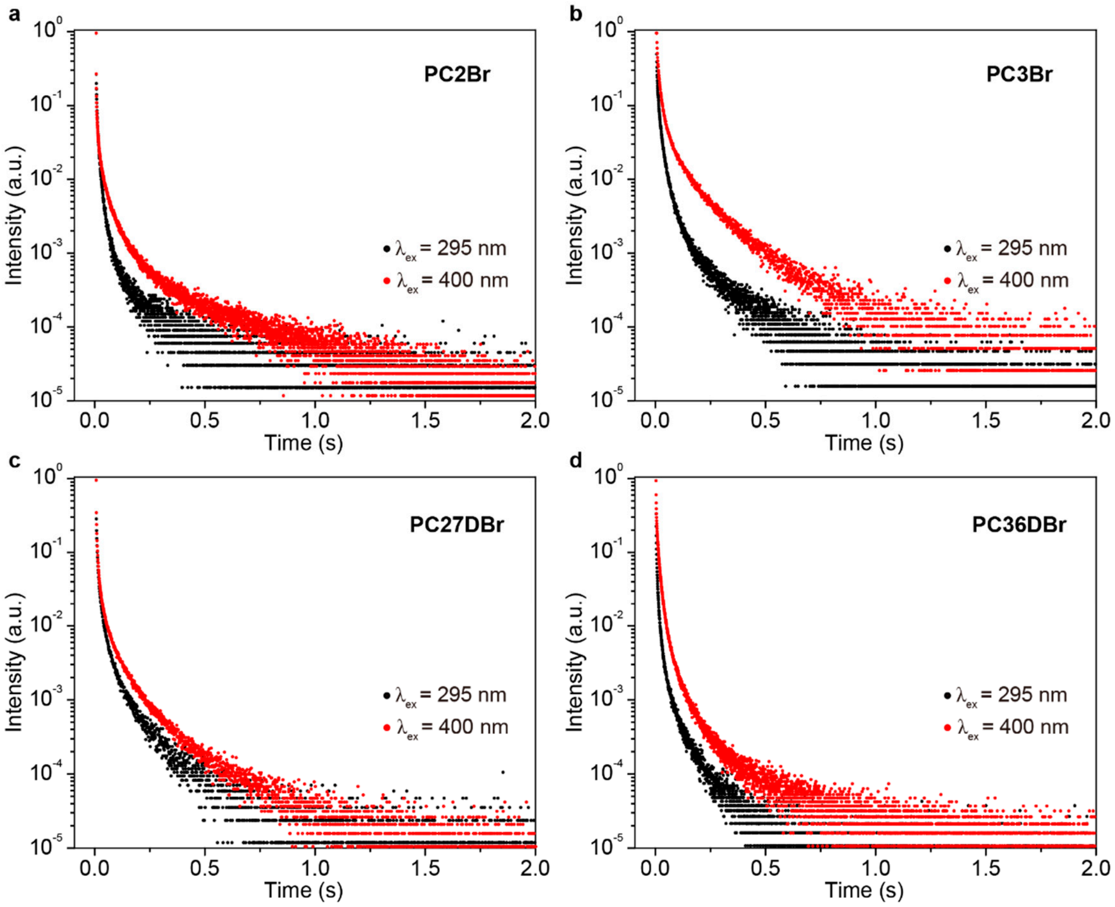

Figure S19. Organic afterglow decay curves of (a) **PC2Br,** (b) **PC3Br,** (c) **PC27DBr** and (d) **PC36DBr** crystals excited at 295 nm and 400 nm at room temperature.

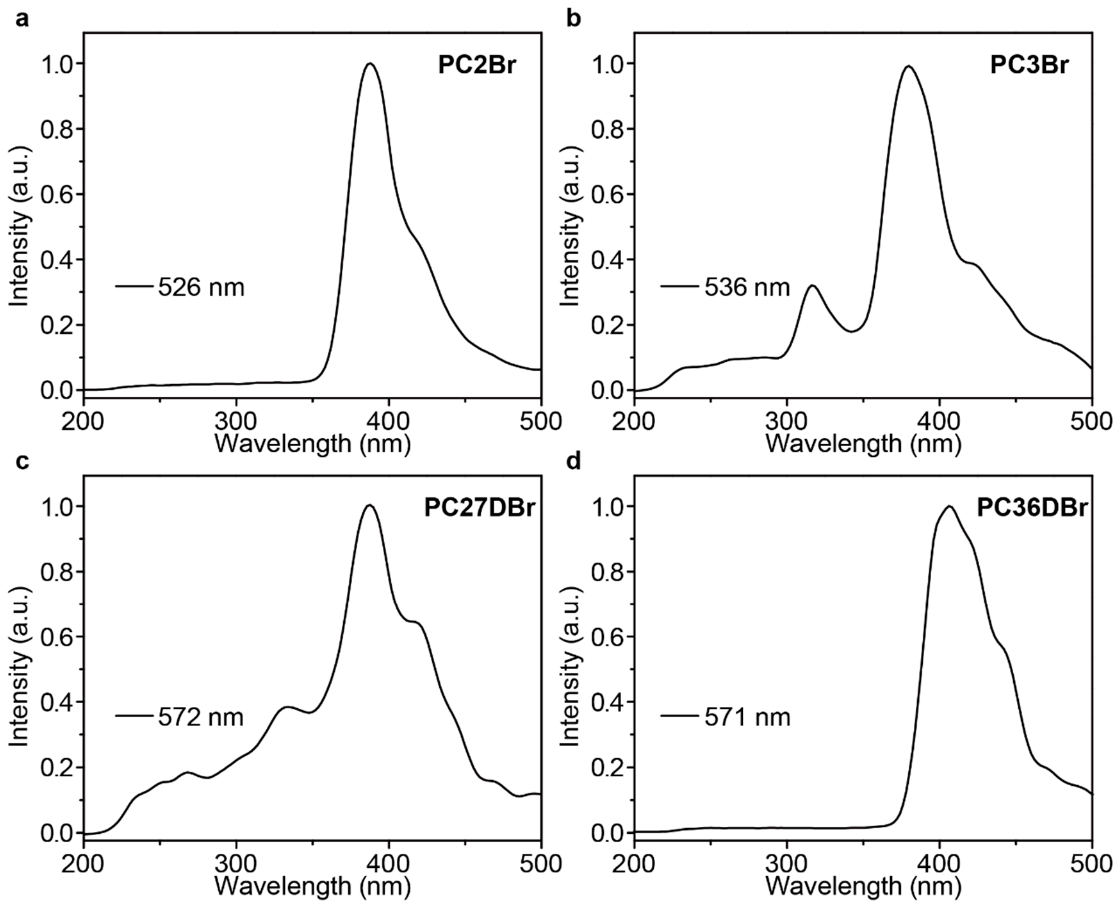

Figure S20. Excitation spectra for organic afterglow peaks of (a) **PC2Br,** (b) **PC3Br,** (c) **PC27DBr** and (d) **PC36DBr** crystals at room temperature.

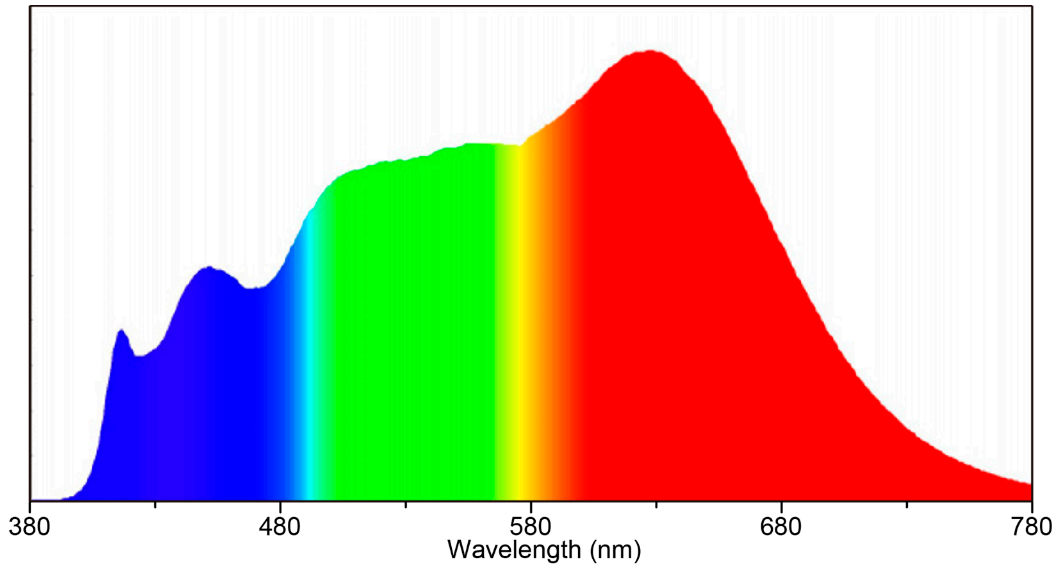

Figure S21. Steady-state emission spectrum of white LED at room temperature.

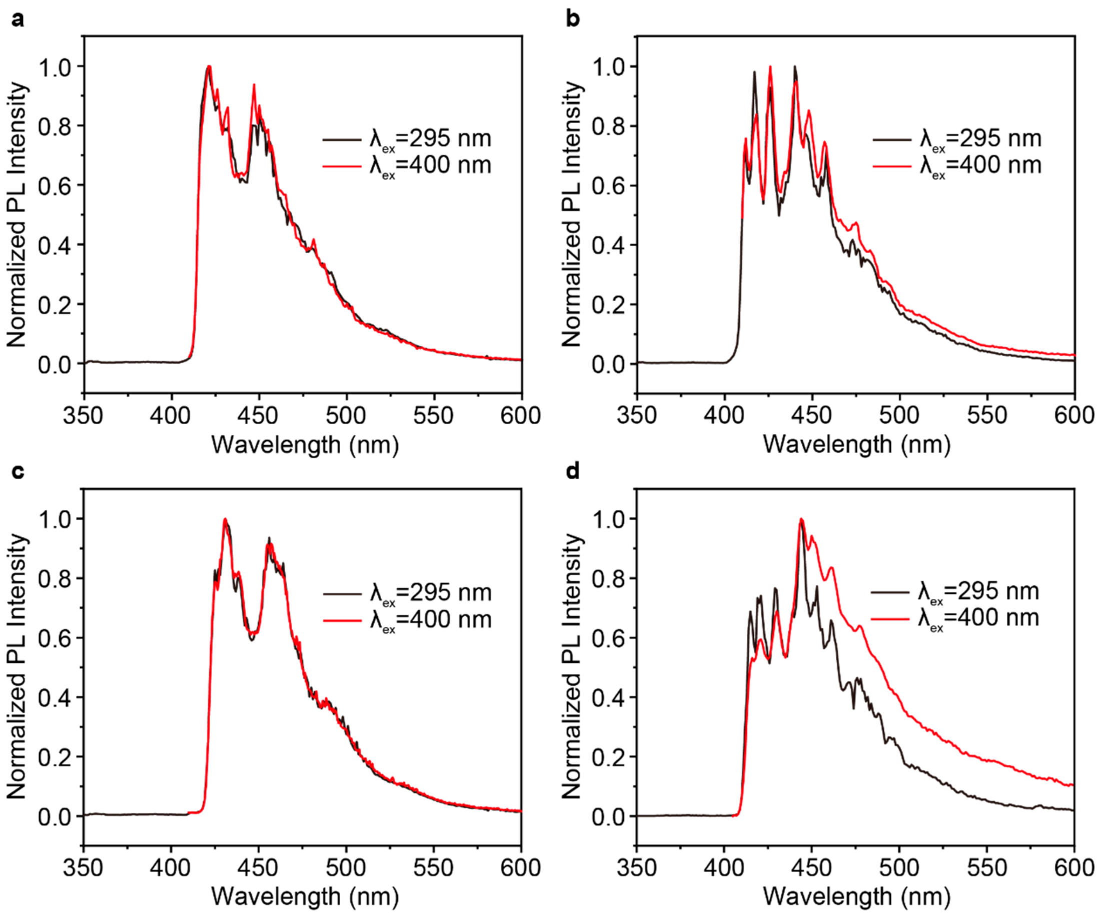

Figure S22. UV-light (295 nm, black line) and visible-light (400 nm, red line) excited phosphorescence spectra of (a) **PC2Br,** (b) **PC3Br,** (c) **PC27DBr** and (d) **PC36DBr** in 2-methyltetrahydrofuran dilute (~10^{-5} mol L^{-1}) solution at 77 K with a delay time of 10 ms.

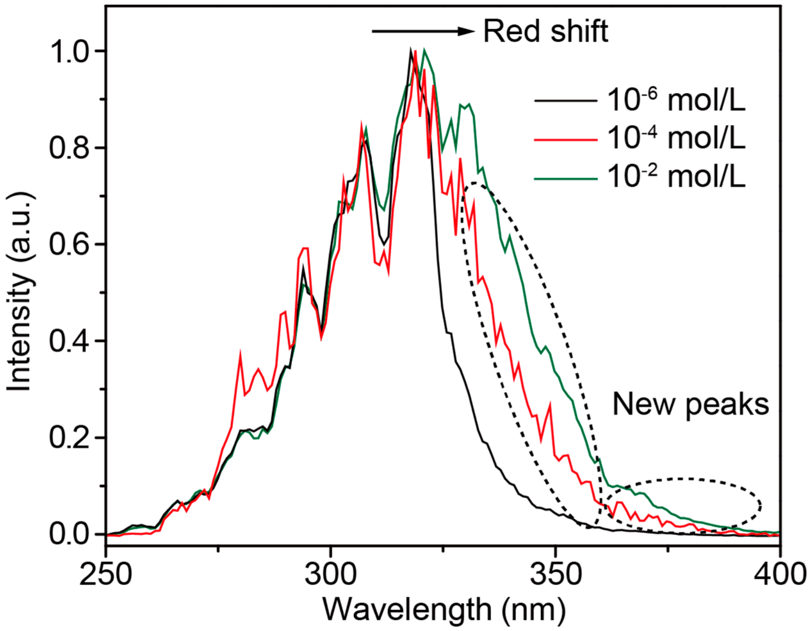

Figure S23. Phosphorescence excitation spectra of **PC2Br** in 2-methyltetrahydrofuran solution with increasing concentration at 77 K, recorded with a delay time of 10 ms.

Probing the formation of triplet excited states

The presence of triplet states in the solutions of Br-substituted 9-phenyl-9H-carbazole (**PhCz)** derivatives was assessed through the photodegradation of 1,3-Diphenylisobenzofuran (**DPBF**). Essentially, the energy transfer from the triplet excited state of the compound to the ground state of molecular oxygen ($^3O_2$) results in the formation of an electronically excited state of molecular oxygen, namely singlet oxygen ($^1O_2$), upon photoexcitation. Thus, **DPBF** serves as a chemical trap to detect generated $^1O_2$, and this trapping process can be quantitatively monitored using a UV-vis spectrophotometer. Specifically, in the presence of triplet states and generated $^1O_2$, the characteristic absorption peaks of **DPBF** (424 nm) gradually diminish in intensity and eventually disappear.

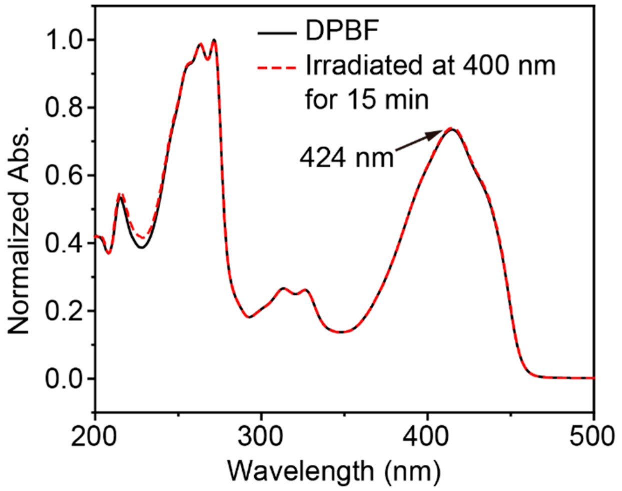

Figure S24. Absorption spectra of **DPBF** (50 μM) in THF under 400 nm irradiation for 15 min at room temperature.

Time-dependent density functional theory (TD-DFT) calculations

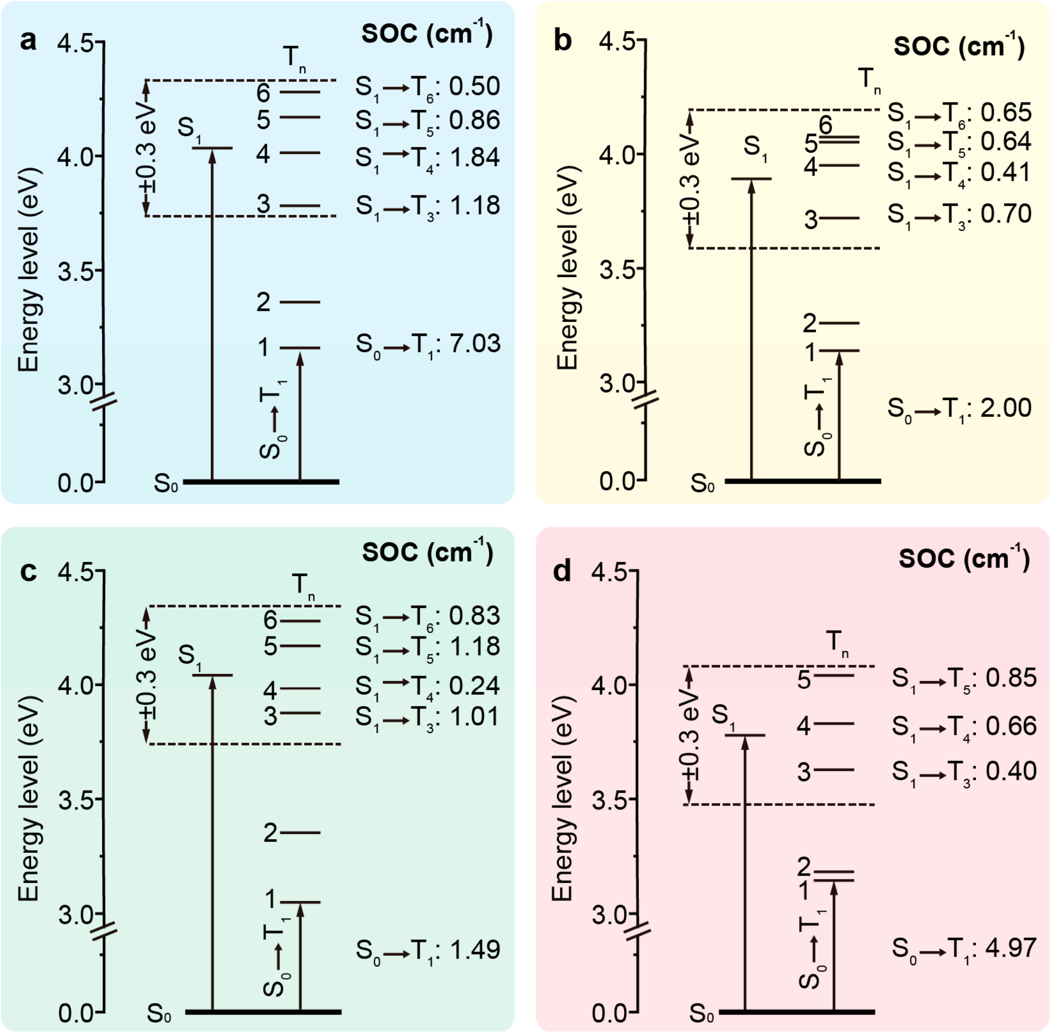

Figure S25. Schematic diagrams showing the TD-DFT-calculated energy levels at singlet (S_n) and triplet (T_n) states and spin-orbit coupling (SOC) values for $S_1 \rightarrow T_n$ and $S_0 \rightarrow T_n$ of (a) **PC2Br,** (b) **PC3Br,** (c) **PC27DBr** and (d) **PC36DBr.** Note that only the most possible T_n with singlet-triplet splitting (ΔE_{ST}) lower than 0.3 eV were illustrated.

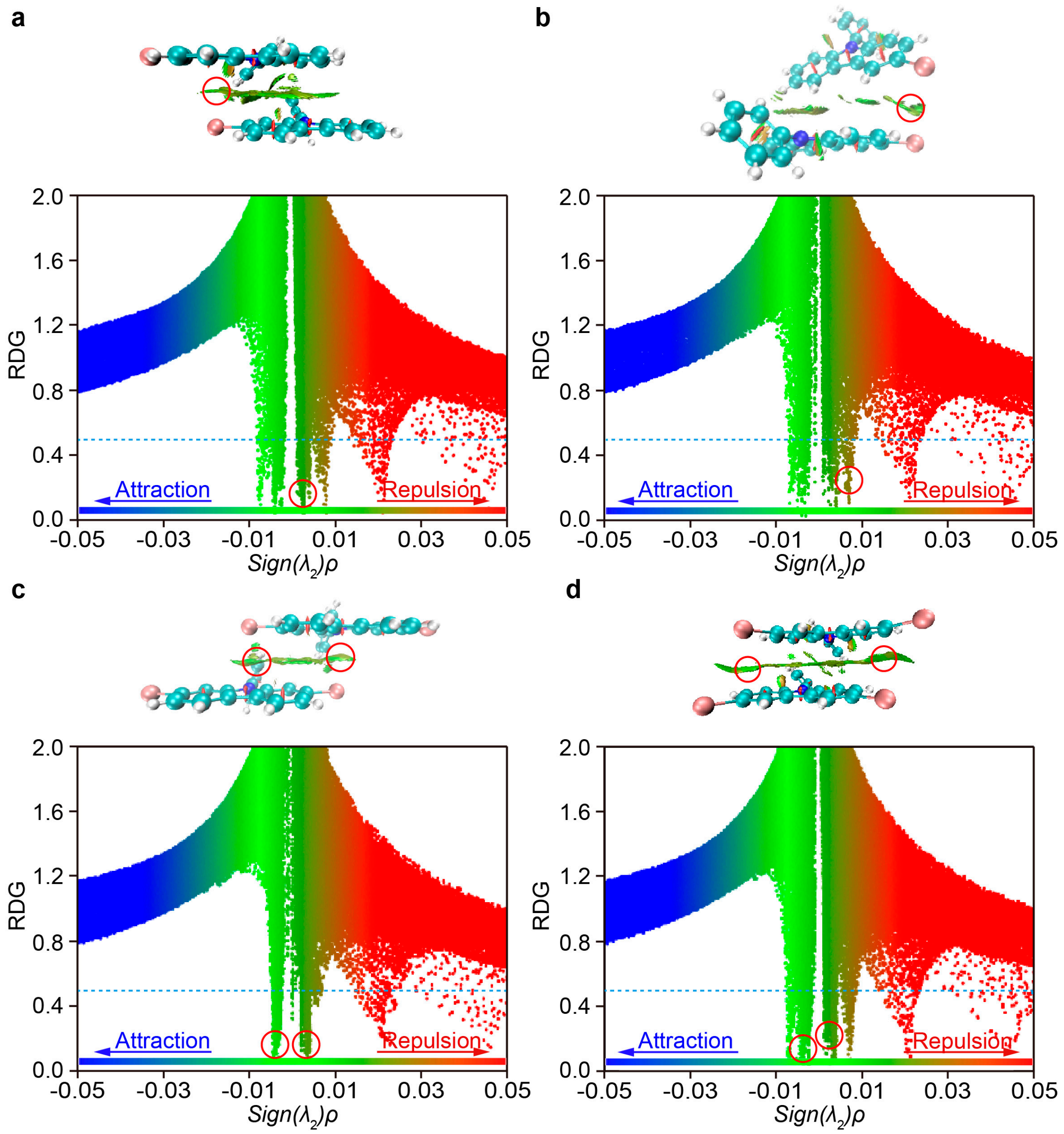

Figure S26. Analysis of intermolecular heavy atom effect. Plots of reduced density gradient (RDG) versus *sign(λ2)ρ* with RDG isosurface of the selected dimers in (a) **PC2Br**, (b) **PC3Br**, (c) **PC27DBr** and (d) **PC36DBr** single crystals. Intermolecular heavy atom interactions are highlighted in red circles.
